# Synthesis and preclinical evaluation of FAP-targeting radiotracers for PET and optical imaging

**DOI:** 10.1186/s41181-025-00398-9

**Published:** 2025-12-05

**Authors:** Jürgen Kogler, Cornelius K. Donat, Johanna Trommer, Klaus Kopka, Sven Stadlbauer

**Affiliations:** 1 Institute of Radiopharmaceutical Cancer Research, Helmholtz-Zentrum Dresden-Rossendorf (HZDR), 01328 Dresden, Germany; 2 Faculty of Chemistry and Food Chemistry, School of Science, Technische Universität Dresden, 01062 Dresden, Germany; 3 German Cancer Consortium (DKTK), Partner Site Dresden, and German Cancer Research Center (DKFZ), 69120 Heidelberg, Germany; 4 National Center for Tumor Diseases (NCT), NCT/UCC Dresden, a Partnership Between DKFZ, Faculty of Medicine and University Hospital Carl Gustav Carus, TUD Dresden University of Technology, and Helmholtz-Zentrum Dresden-Rossendorf (HZDR), 01307 Dresden, Germany

**Keywords:** FAP, PET, Fluorescence-guided surgery, Noninvasive molecular imaging

## Abstract

**Background:**

Successful treatment of solid cancers relies on precise diagnosis, e.g. using noninvasive molecular imaging, followed by surgical removal and/or chemo/immunotherapy. Despite advances in pre-operative imaging, real-time intraoperative tools remain limited, which often results in high rates of tumor-positive margins and recurrence after tumor resection. To address this limitation, we aimed to develop multifunctional fibroblast activation protein alpha (FAP) targeting tracers for bimodal medical imaging, enabling both pre-operative noninvasive molecular imaging via positron emission tomography (PET) and optical visualization during intraoperative fluorescence-guided surgery.

**Results:**

NODAGA-FAP647 and NODAGA-FAP800 targeting human FAP (hFAP) were synthesized bearing a (R)-NODAGA chelator and a fluorophore (AlexaFluor647 or IRDye800CW, respectively). Binding affinities and binding kinetics of both unlabeled and ^67/68^Ga-labeled compounds were evaluated in vitro using HT1080 cells (hFAP-expressing and wild type, WT) along with respective frozen xenograft tissue sections. Using real-time binding, both compounds exhibited picomolar binding affinities to hFAP via radioactive/fluorescent detection. This was primarily driven by low dissociation rate constants in vitro. Pharmacokinetics and tumor uptake were evaluated via PET and fluorescence imaging in mice bearing xenografts from the same cells. In vivo, both compounds were rapidly distributed and accumulated in hFAP-expressing but not WT-HT1080 tumors within 10–20 min post-injection. Fluorescence imaging showed a similarly good and selective tumor uptake in the first two hours and a qualitatively visible difference compared to WT-HT1080 beyond 24 h. Both compounds were quickly cleared from normal tissue and excreted renally.

**Conclusion:**

Two FAP-targeting bimodal ligands were synthesized and evaluated in vitro and in vivo, showing high specificity and selectivity, along with rapid and selective tumor accumulation. Their long tumor retention and high imaging contrast make them promising candidates for clinical translation.

**Supplementary Information:**

The online version contains supplementary material available at 10.1186/s41181-025-00398-9.

## Background

Successful treatment of solid cancers requires several tightly linked procedures: diagnosis, surgical resection and/or radio/chemo/immunotherapy. Among other diagnostics, noninvasive molecular imaging, such as positron emission tomography (PET) and single photon emission computed tomography (SPECT) play an important role. Several new biomarkers, such as prostate-specific membrane antigen (PSMA) and fibroblast activation protein (FAP, seprase or FAPα) along with their respective radiotracers have recently allowed a faster and more precise detection of several solid cancers (Schwenck et al. 2023). These diagnostic advances facilitate better planning of the next treatment step: surgical resection, which is still the first-line therapy for most solid tumors (Are et al. 2024). However, far less tools are available to support the surgeon during the actual resection. There, removing the tumor with a sufficient tumor-free margin is key, while simultaneously preserving the healthy surrounding tissue (Lauwerends et al. 2021; Koller et al. 2018; Rosenthal et al. 2016). However, the incomplete surgical removal represents a prevalent course for the reoccurrence of cancer to date (Hiller et al. 2018). The margins of the resected tissue are typically analyzed by histopathological methods peri- or post-operatively, e.g. by using (frozen) tissue sections (Lauwerends et al. 2021; Hernot et al. 2019). This can be time-consuming and prone to false positive/negative results, as only a small fraction of the specimen can be subjected to analysis (St John et al. 2017; Cendan et al. 2005). The rates of tumor-positive margins of the resected tissues can reach up to 70% dependent on the cancer entities (Hernot et al. 2019; Tilly et al. 2014; Wong et al. 2012; Esposito et al. 2008; Vos et al. 2017). Consequently, the resection success depends mainly on the surgeon’s experience, training and tactile information, allowing to distinguish between healthy and malignant tissues (Rosenthal et al. 2016, 2015). To support the surgeon, real-time guidance techniques, e.g. intraoperative fluorescence-guided surgery, were recently introduced into clinical practice (Lauwerends et al. 2021; Sutton et al. 2023). Due to the high sensitivity and spatial resolution, optical fluorescence imaging ideally complements pre-operative diagnostics such PET, SPECT, CT and/or MRI (Kubeil et al. 2022; Ariztia et al. 2022). The application of fluorophores with a maximum emission wavelength in the near-infrared (NIR) region is considered advantageous. This is mainly due to the increased range in tissue and penetration, along with reduced effect of auto-fluorescence, ultimately resulting in higher sensitivity and resolution (Cheong et al. 1990; Gioux et al. 2010; Keereweer et al. 2013).

A number of non-targeted fluorescent probes, such as indocyanine green (ICG), methylene blue (MB) and 5-aminolevulinic acid (5-ALA) are used in clinical practice, with new probes targeting specific biomarkers being developed (Koller et al. 2018; Stummer et al. 2006; Zvinys et al. 2024; Odenthal et al. 2018; Baranski et al. 2018; Manen et al. 2018). Among those, FAP became a prominent target in recent years, especially for theranostic radiotracers (Millul et al. 2023; Serumula et al. 2025; Imlimthan et al. 2021; Desaulniers et al. 2025). The enzyme is involved in a wide range of physiological and pathological processes via the cleavage of bioactive peptides (Gorrell 2005). In an oncological context, FAP is mainly expressed in the tumor microenvironment (TME) on cancer associated fibroblasts (CAFs) (Hamson et al. 2014). In 2014, a series of highly potent and selective inhibitors was reported by Van der Veken and coworkers, bearing a quinoline motif and a nitrile warhead moiety (Jansen et al. 2013, 2014; Poplawski et al. 2013). Based on these structures, a series of radiotracers were developed by Haberkorn and coworkers, with FAPI-04 and FAPI-46 being the most promising compounds (Lindner et al. 2018; Loktev et al. 2019). These diagnostic radiotracers exhibited high tumor accumulation, a rapid clearance from healthy tissue and fast renal excretion (Kratochwil et al. 2019). Based on these, we aimed to develop a single multifunctional compound for bimodal imaging to be utilized for both pre-operative diagnostics and during intraoperative fluorescence-guided surgery. The utilization of bimodal imaging agents offers a distinct advantage, as their pharmacodynamics and kinetics are identical for both imaging modalities. This characteristic facilitates co-localization prior to and during the surgical intervention, which can vary for two different imaging agents (Louie 2010; Minges et al. 2025).

In order to design FAP-targeting bimodal imaging agents, the fluorophore (IRDye800CW or AlexaFluor647) and the chelator (R)-NODAGA were conjugated to the FAP inhibitor. Subsequently, a biological evaluation of the ligands was conducted. This evaluation included the determination of binding affinity towards FAP, as well as assessments of stability and pharmacokinetic profile in vivo.

## Results

### Chemistry and radiochemistry

Intermediate **8** was synthesized following adapted procedures from the literature of the small-molecule inhibitor UAMC1110 and FAPI-46 (Supplementary Fig. 1) (Jansen et al. 2014; Loktev et al. 2019). To achieve a straightforward and cost-efficient synthesis, an l-lysine amino acid was chosen as central linker structure. The orthogonally *N*-Fmoc- and *N´*-Boc-protected amino acid l-lysine was coupled to the secondary piperazinyl amine of **8**. The chelator (R)-NODAGA, protected as tris *tert*-butyl ester, was coupled to the ε-amine of the lysine motif after Boc deprotection. Subsequently, a tetraethylene glycol (PEG_4_) spacer was introduced via an amide coupling reaction to the α-amine, yielding the central intermediate building block **13** after Fmoc deprotection. The PEG_4_ spacer was introduced to create spatial distance between the FAP-binding motif and the fluorescent dye, to avoid steric hindrance and interference with binding to the active site of FAP. AlexaFluor647 and IRDye800CW, in the form of their *N*-hydroxysuccinimidyl activated esters were conjugated to the primary amine of **13** via another amide coupling, yielding NODAGA-FAP647 **14** and NODAGA-FAP800 **15** with an overall yield of 4% over 10 steps for both derivatives (Supplementary Fig. 2). The precursors were radiolabeled with ^68^Ga resulting in molar activities of 27.9 ± 10.1 MBq/ nmol (*n* = 10), and 14.8 ± 6.63 MBq/ nmol (*n* = 10) for ^67^Ga, respectively.

### Determination of the log ***D***_7.4_

The hydrophilicity of the ^68^Ga-labeled radiotracers was determined, reflected by the log *D*_7.4_ value, using the shake-flask method. [^68^Ga]Ga-NODAGA-FAP647 and [^68^Ga]Ga-NODAGA-FAP800 exhibited high hydrophilicity with log *D*_7.4_ values of – 4.10 ± 0.08 and – 3.54 ± 0.21, respectively. The hydrophilicity was greater than that of the diagnostic radiotracer [^68^Ga]Ga-FAPI-46 (log *D*_7.4_: – 3.38 ± 0.01). This is likely attributed to the four to five sulfonic acid groups of the fluorescent dyes that are deprotonated at physiological pH. This results in an overall negative charge of – 3 to – 4 for the radiotracers.

### Assessment of inhibitory affinity towards FAP and DPPIV

**Fig. 1:**
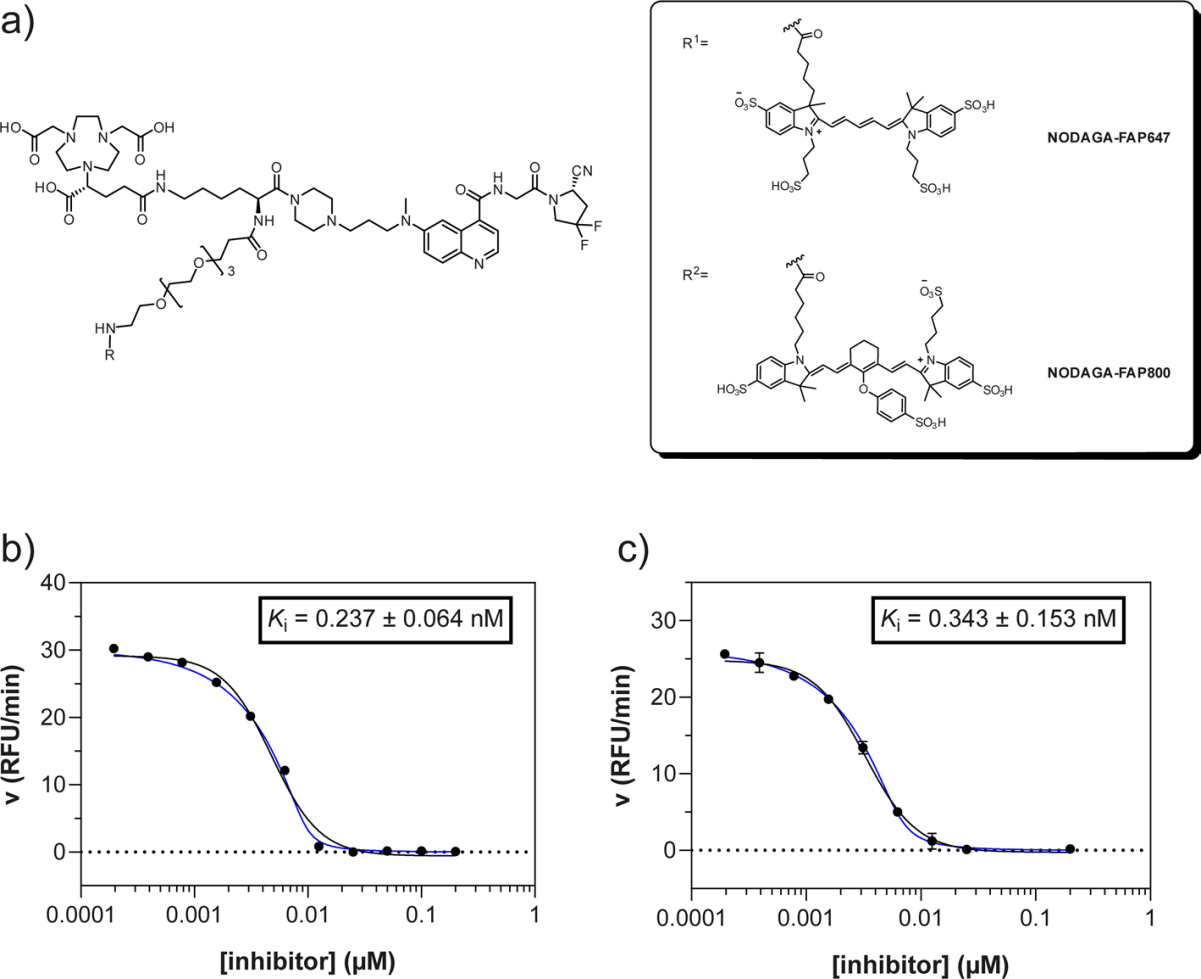
**a** Structures of multifunctional FAP inhibitors, with the inhibitor moiety UAMC1110, chelator (R)-NODAGA and the fluorescent dye AlexaFluor647 (NODAGA-FAP647) or IRDye800CW (NODAGA-FAP800). Inhibition of human recombinant FAP with NODAGA-FAP647 **b** or NODAGA-FAP800 **c** as a single experiment, which was performed in duplicate. Black line represents fit by non-linear regression analysis equation for IC_50_, blue line represents fitting by the Morrison equation for tight binding inhibitors; Values are mean ± SD

Both NODAGA-FAP647 and NODAGA-FAP800 showed a concentration-dependent inhibition of hFAP, which could be seen by a reduction of the conversion rate of the fluorogenic substrate Ala-Pro-AMC in a linear fashion (Fig. 1b and c, Supplementary Fig. 9). The IC_50_ values, which were determined from the dose–response curves, were found to be in the same range as the enzyme concentration of 7.25 nM that was utilized in the assay. In this particular case, the IC_50_ value would be underestimated, because it would always diverge towards half of the enzyme concentration, as shown in the equation below (Copeland 2013).

$$ {\text{IC}}_{{{5}0}} = K_{{\text{i}}} + \, \raise.5ex\hbox{$\scriptstyle 1$}\kern-.1em/ \kern-.15em\lower.25ex\hbox{$\scriptstyle 2$} *\left[ {\text{E}} \right] $$

For such high-affinity ligands, the *K*_*i*_ can be determined using the Morrison equation for tight binding inhibitors, which takes the concentration of active enzyme into account (Williams and Morrison 1979). Using the Morrison equation, picomolar *K*_i_ values of 0.237 ± 0.064 nM and 0.343 ± 0.153 nM were determined for NODAGA-FAP647 and NODAGA-FAP800, respectively (Fig. 1b and c).

The selectivity towards dipeptidyl peptidase IV (DPPIV) was tested, due to the shared sequence homology to FAP of 54% (Levy et al. 1999) and the ubiquitous expression of DPPIV (Mortier et al. 2016). The IC_50_ was determined using a fluorometric enzyme inhibition assay. IC_50_ values were found to be higher than 10 μM for both NODAGA-FAP647 and NODAGA-FAP800 (Supplementary Fig. 10), resulting in a selectivity of over 29,000 for FAP over DPPIV.

### Saturation binding of [^68^Ga]Ga-NODAGA-FAP647 and [^68^Ga]Ga-NODAGA-FAP800 to hFAP-HT1080 cells

**Fig. 2:**
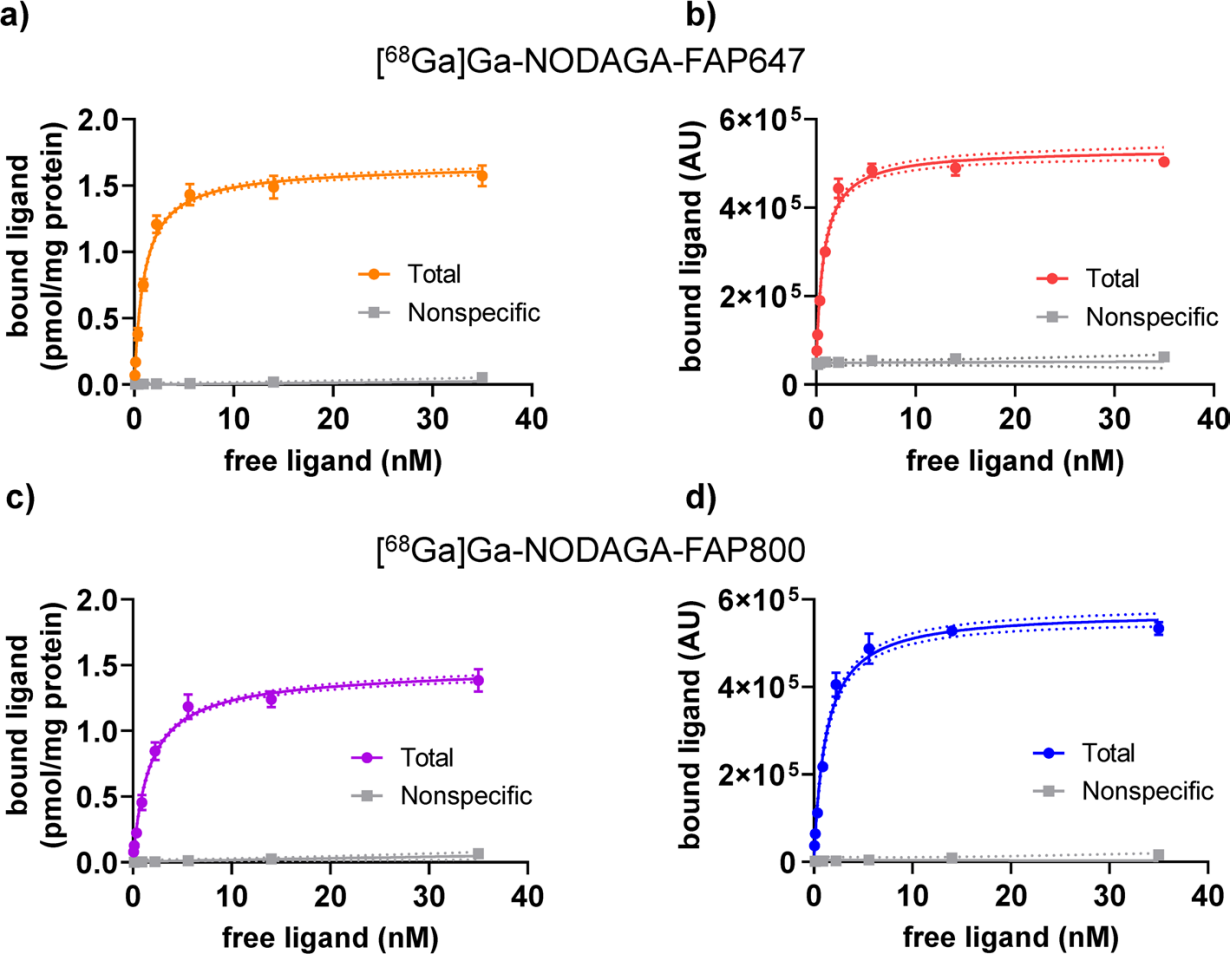
Saturation binding of [^68^Ga]Ga-NODAGA-FAP647 (upper row) and [^68^Ga]Ga-NODAGA-FAP800 (lower row) to hFAP-HT1080 cells. Nonlinear iterative curve fitting derived from radioactive (**a/c**, *n* = 3) and fluorescent detection (**b/d**, *n* = 1). Total binding is represented by the solid-colored lines (95% confidence intervals as dotted lines), grey lines represent nonspecific binding in the presence of 14 µM FAPI-04

For the assessment of the binding in a cellular context, saturation binding experiments on HT1080 cells, stably transduced to express hFAP (hFAP-HT1080), were performed. The equilibrium constant *K*_D_ and the maximum number of binding sites *B*_max_ were determined via detection of the radioactive/fluorescent signal of the ^68^Ga-labeled radiotracers (Fig. 2). Non-specific binding was determined in the presence of 14 µM FAPI-04. Binding of both compounds to hFAP-HT1080 cells was saturable and efficiently blocked in the presence of 14 µM FAPI-04. Nonlinear iterative curve fitting yielded low nanomolar *K*_D_ values, with comparable results for radioactivity or fluorescence detection (Fig. 2, Table 1). The *B*_max_ was similar for both compounds and across experiments. When fluorescence of the compounds was used as method of detection, curve fitting resulted in slightly lower *K*_D_ values (~ 25%) and, again, similar *B*_max_ values (Table 1)_._ The data indicates that the concentration range could potentially benefit from a shift towards lower concentrations. However, radioactivity and fluorescence in the lowest concentrations (143 and 57 pM, respectively) were approaching the respective detection thresholds, hence this was not further explored.

**Table 1:** *K*_D_ and *B*_max_ for [^68^Ga]Ga-NODAGA-FAP647 and [^68^Ga]Ga-NODAGA-FAP800 from saturation binding experiments

	Radioactive label detection (*B*_max_: (pmol/mg/protein))		Fluorescent label detection (*B*_max_: (AU))	
[^68^Ga]Ga-NODAGA-FAP647	Best fit	95% CI	Best fit	95% CI
*B*_max_	1.63	1.60 – 1.66	4.8 * 10^5^	4.6 – 4.9 * 10^5^
*K*_D_ (nM)	0.99	0.92 – 1.07	0.76	0.66 – 0.87

[^68^Ga]Ga-NODAGA-FAP800				
*B*_max_	1.41	1.38 – 1.45	5.7 * 10^5^	5.5 – 5.9 * 10^5^
*K*_D_ (nM)	1.65	1.49 – 1.81	1.24	1.08 – 1.42

### Real-time binding of ^67/68^Ga-labeled NODAGA-FAP647 and NODAGA-FAP800 with radioactive and fluorescent detection

For a more detailed evaluation of the binding kinetics, a real-time binding assay was performed in vital target-positive and negative cells, target positive frozen xenograft tissue sections along with various control experiments. In this assay, association rate constant *k*_a_ and the dissociation rate constant *k*_d_ are determined for both compounds, yielding *K*_D_. Again, radioactivity (^67/68^Ga-labeling) and fluorescence were both used for signal detection.

**Fig. 3:**
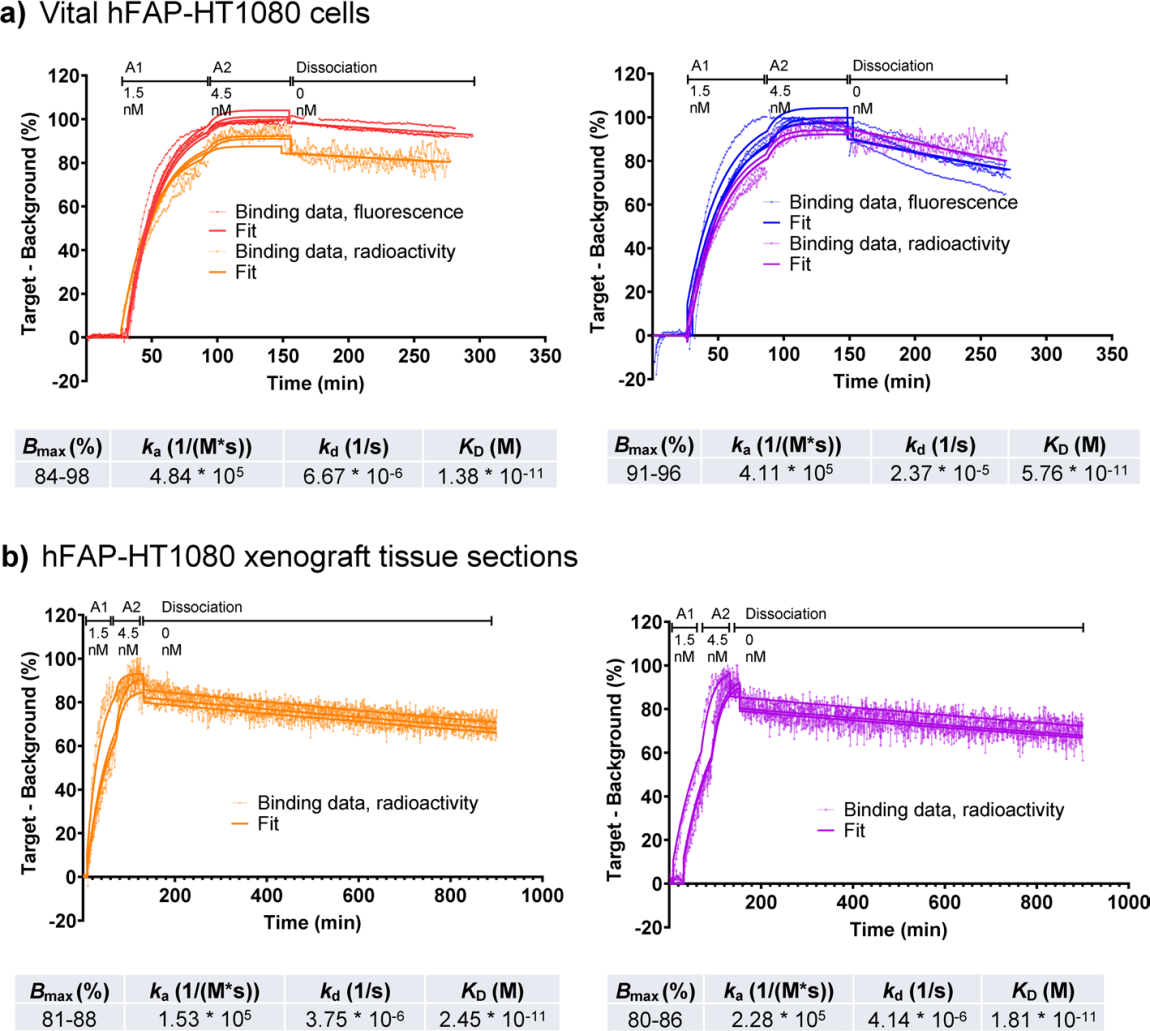
**a** Real-time binding of [^68^Ga]Ga-NODAGA-FAP647 (left) and [^68^Ga]Ga-NODAGA-FAP800 (right) to vital hFAP- HT1080 cells. Data indicates tight binding/slow dissociation of both compounds. **b** Binding of [^67^Ga]Ga-NODAGA-FAP647 (left) and [^67^Ga]Ga-NODAGA-FAP800 (right) to 25 µm frozen tissue sections, derived from hFAP-HT1080 xenografts. Data confirms slow dissociation in fresh-frozen tissue sections. Real-time binding experiments were performed on hFAP-HT1080 cells (*n* ≥ 6). Values derived from a global fit of association and dissociation rate constant and resulting *K*_D_. The U-value describes the variation (in %) before the fitted results are significantly changed. It provides a better accuracy measure than the standard error of the mean, as it takes parameter dependency into account. Maximum number of binding sites (*B*_max_) is fitted for each individual curve, and the range is given. Each trace was normalized to baseline (0%) and highest value (typically at the end of the second association phase, 100%). This approach allows global fits without signal intensity differences, e.g. caused by varying molar activities. Colored lines with symbols (red/orange: fluorescent/radioactive label detection of [^68^Ga]Ga-NODAGA-FAP647; blue/purple: fluorescent/radioactive label detection of [^68^Ga]Ga-NODAGA-FAP800) represent individual traces (actual binding, signal of target − background area in %; each decay or fluorescence corrected), with fitted data indicated by respective colored solid lines. Kinetic constants were fitted over all traces (global), while *B*_max_ was fitted per binding curve (local). Data from *n* = 6/7/3 independent experiments using either radioactive or fluorescent label detection

Real-time binding, using live cells, revealed a fast association along with a rather slow and linear dissociation process, resulting in low dissociation rate constants (Fig. 3a, Supplementary Fig. 11). The latter was confirmed over a longer (five hours) dissociation period (Supplementary Fig. 12). Due to the stable binding, picomolar binding affinities of 14 pM and 58 pM were determined for [^68^Ga]Ga-NODAGA-FAP647 and for [^68^Ga]Ga-NODAGA-FAP800, respectively (Fig. 3, Table 2).

**Table 2:** Kinetic parameters derived from fitting real-time binding of [^68^Ga]Ga-NODAGA-FAP647 and [^68^Ga]Ga-NODAGA-FAP800

Best fit	*B*_max_ (%)	*k*_a_ (1/(M*s))	*k*_d_ (s^−1^)	*K*_D_ (M)	U-value (%)
[^68^Ga]Ga-NODAGA-FAP647	84–98	4.84 * 10^5^	6.67 * 10^−6^	1.38 * 10^−11^	12.80
[^68^Ga]Ga-NODAGA-FAP800	91–96	4.11 * 10^5^	2.37 * 10^−5^	5.76 * 10^−11^	8.10

When fluorescence was detected instead of radioactivity, the kinetics for [^68^Ga]Ga-NODAGA-FAP647 did not differ (Supplementary Fig. 11a). In contrast, fluorescent detection of NODAGA-FAP800 yielded faster association (~ 50%) and also faster dissociation (~ 30%) kinetics compared to radioactive detection (Supplementary Fig. 11b). This might point to different stabilities of the two fluorophores over time or other effects, such as photobleaching.

In real-time binding, substantial internalization of a tracer would result in a compounded binding affinity measure, as dissociation kinetics would include dissociation of the membrane-bound tracer and efflux processes of the internalized fraction. This would also result in low dissociation rate constants and, in turn, an overestimation of *K*_D_. To circumvent this, sections prepared from fresh-frozen hFAP-HT1080 xenograft tissue were used. These do not contain living cells, and the freezing process typically leads to membrane perforation from quickly forming ice crystals, which makes internalization highly unlikely. Labeling was carried out with ^67^Ga due to the longer half-life of 78.3 h and dissociation was monitored for over 10 h. Binding of both ^67^Ga-labeled compounds to tissue sections exhibited the same pattern observed in cells, i.e. a fast association and a slow and linear dissociation (*k*_d_ between 10^−5^ – 10^−6^ s^−1^, Fig. 3b). As internalization is highly unlikely, this indicates the formation of a very stable enzyme-inhibitor complex and implies a tight binding behavior, as seen in the fluorometric enzyme inhibition assay. Overall, binding kinetics in tissue sections were comparable to living cells, yielding similar *K*_D_ values (25 pM for [^67^Ga]Ga-NODAGA-FAP647 and 18 pM for [^67^Ga]Ga-NODAGA-FAP800). Differences are likely to be attributed to diffusion processes taking place in the tissue sections, in contrast to a more freely accessible monolayer of cells.

A number of control experiments were conducted to probe target specificity and selectivity, along with additional experiments to confirm the stable binding. Respective experimental details are provided in the Supplementary Information. Target specificity was confirmed using wild type HT1080 cells, lacking any substantial FAP expression. Neither [^68^Ga]Ga-NODAGA-FAP647 nor [^68^Ga]Ga-NODAGA-FAP800 produced any signal increase (slopes close to zero) over time, hence lacking any binding (Supplementary Fig. 13a). Target selectivity was investigated using a structurally different FAP-inhibitor, FAPI-04. A twenty-time excess of FAPI-04 was added at the end of the 2nd association phase, with the respective ligand still present. This excess of cold competitor was unable to significantly displace the already bound NODAGA-FAP647/800 (Supplementary Fig. 13b). The curve slopes of the displacement phase were similar to those observed during dissociation (Supplementary Fig. 13b), indicating that FAPI-04 can only bind at the rate of NODAGA-FAP647/800’s dissociation. Binding pattern and kinetics of NODAGA-FAP647/800 to PFA-fixed hFAP-HT1080 cells were similar (Supplementary Fig. 14a) to that observed in live cells and tissue sections. An eight-minute wash with glycine buffer (pH = 2.8) was unable to remove any bound tracer and resulted in similar dissociation rate constants as observed in cells (Supplementary Fig. 14b). These findings further corroborate a very stable target-ligand complex and therefore a slow dissociation process, rather than any internalization.

### Cellular binding pattern as imaged by fluorescence microscopy

**Fig. 4:**
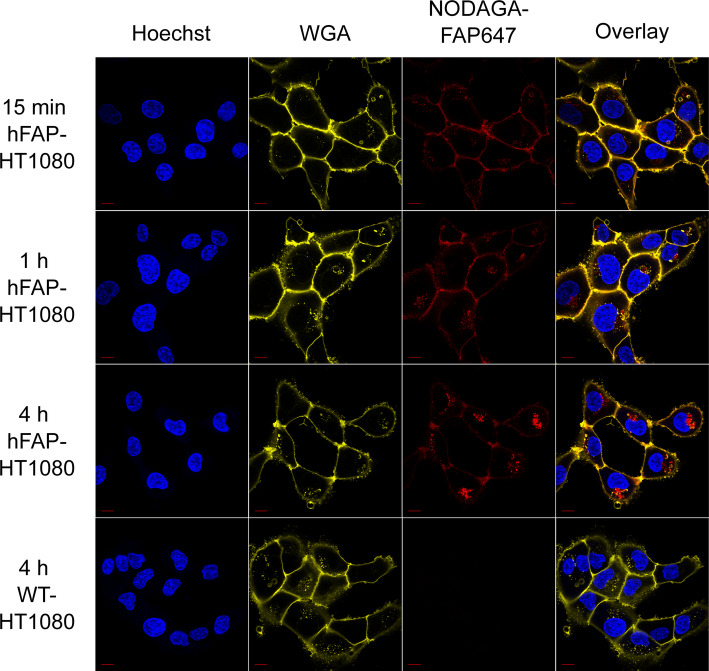
Representative images of NODAGA-FAP647 binding to hFAP-HT1080, along with WT-HT1080 cells as control. Cells were incubated with NODAGA-FAP647 (red) for 15 min, 1 h and 4 h, afterwards the cells were fixed with PFA and stained with Hoechst for cell nuclei (blue) and WGA-CF488A for cell membranes (yellow), Scale bars represent 20 µm

The cellular binding pattern and uptake was investigated using fluorescence microscopy with NODAGA-FAP647 on hFAP-HT1080 cells, followed over a four-hour period at 37 °C. Throughout this time, intrinsic fluorescence, associated with the ligand, was observed at the cell membrane. Over time, this signal gradually decreased but did not vanish. At the same time, fluorescence associated with NODAGA-FAP647 inside the cells increased (Fig. 4). Consistent with that, the fluorescent signal of the ligand accumulated in cellular compartments that are WGA-positive as well. Considering WGA stains the Golgi-complex (Hiller and Weber 1982), this could indicate uptake into lysosomal vesicles. Additionally, a fraction of the ligand-associated fluorescence was found to bind adjacent to the cell nuclei, which was unexpected. Again, this signal co-localized with WGA, which could point to endoplasmic uptake and potentially degradation.

WT-HT1080 cells did not show any binding or uptake (Fig. 4), hence it can be concluded that binding is target-specific, and the uptake is FAP-mediated. While this may seem to contrast with our real-time binding data, substantial differences in both experimental details need to be considered: (1) a much higher concentration of the ligand (1 µM vs 1.5/4.5 nM in real-time binding experiments), (2) a much longer incubation period (4 h for substantial internalization to occur) and (3) a higher temperature (37 °C vs. 21 °C in real-time binding experiments). These differences may provide conditions that favor internalization.

### Stability towards human serum

The stability towards human serum proteins was assessed for both ^67^Ga-labeled radiotracers. Incubation of [^67^Ga]Ga-NODAGA-FAP647 and [^67^Ga]Ga-NODAGA-FAP800 in human serum at 37 °C showed no degradation at the four-hour time point (Supplementary Fig. 8). Degradation for both radiotracers was observed after 24 h, resulting in a fraction of intact tracer of 56% and 22% for [^67^Ga]Ga-NODAGA-FAP647 and [^67^Ga]Ga-NODAGA-FAP800, respectively (Supplementary Fig. 8).

### In vivo PET imaging

**Fig. 5:**
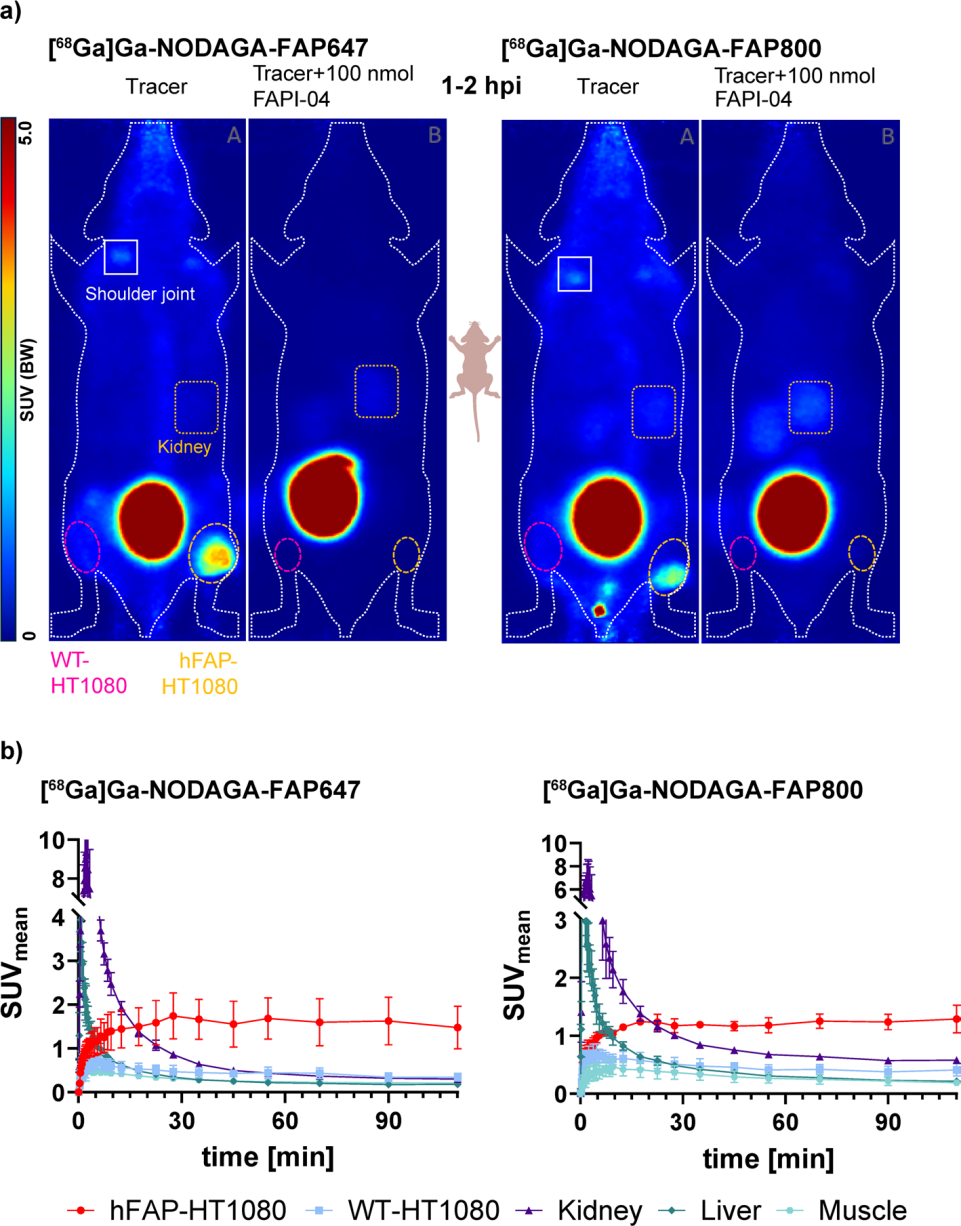
**a** Small animal PET images (Maximum intensity projection) of [^68^Ga]Ga-NODAGA-FAP647 (left) and [^68^Ga]Ga-NODAGA-FAP800 (right) in female NMRI-nude mice (*n* = 4 total for each compound). Relevant volumes of interest (VOIs) are indicated. **b** Time-activity curves (0–2 hpi) of selected VOIs of animals injected with tracer only

Small-animal PET imaging with [^68^Ga]Ga-NODAGA-FAP647 and [^68^Ga]Ga-NODAGA-FAP800 demonstrated a rapid distribution and quick uptake of the tracer in the hFAP-HT1080 tumors (Fig. 5a). Both compounds showed a comparable uptake into hFAP-HT1080 tumors with SUV_max_ values between 2.4 and 3 (Supplementary Fig. 15, 16), with that of [^68^Ga]Ga-NODAGA-FAP647 being slightly higher. Thirty minutes post-injection, the SUV_max/mean_ in the hFAP-HT1080 tumor reached a maximum and showed no washout up to 2 h (Supplementary Fig. 15, 16). A good contrast between hFAP-HT1080 and WT-HT1080 xenografts was observed, from 10 min post-injection onwards (Fig. 5b, Supplementary Fig. 15, 16, Supplementary Table 1). Radioactivity in hFAP-HT1080 xenografts was 236% (SUV_mean_, [^68^Ga]Ga-NODAGA-FAP800) to 392% (SUV_max_, [^68^Ga]Ga-NODAGA-FAP647) higher than in the corresponding WT-HT1080 tumor.

The difference between both tumor entities (Supplementary Fig. 15) was statistically substantiated by a two-way ANOVA, which indicated a significant main effect of SUV_mean_ for both tumor entity [F (1, 4) = 11.19; *P* = 0.0287] and time [F (1.331, 5.324) = 18.01; *P* = 0.0057] for [^68^Ga]Ga-NODAGA-FAP647. Furthermore, there was significant interaction of tumor entity and time [F (34, 136) = 10.41; *P* < 0.0001]. The same was observed for [^68^Ga]Ga-NODAGA-FAP800 with tumor entity [F (1, 4) = 24.63; *P* = 0.0077], time [F (1.731, 6.923) = 45.96; *P* = 0.0001] and a significant interaction [F (34, 136) = 21.27; *P* < 0.0001]. Ex vivo autoradiography and fluorescence imaging of [^68^Ga]Ga-NODAGA-FAP647 showed an identical distribution pattern of the radioactive and fluorescent signal in the hFAP-HT1080 tumor sections (Supplementary Fig. 19).

Both compounds were rapidly cleared, primarily through the kidneys and accumulation of radioactivity in the bladder (Fig. 5a/b, Supplementary Fig. 16). Some uptake into the liver can be observed, which also cleared quickly. It is less likely to be related to just blood flow, as muscle tissue does not exhibit the same overall pattern of fast uptake and washout (Fig. 5b). Both compounds also accumulated in the main joints, primarily in the shoulder. This pattern was also demonstrated by other FAP-targeting tracers previously, both in animals and humans (Hu et al. 2022; Matsusaka et al. 2024).

Specificity towards FAP of both compounds was demonstrated by pre-injection of 100 nmol FAPI-04 resulting in an almost complete lack of uptake (> − 90%, Supplementary Fig. 16) in the hFAP-HT1080 tumor for [^68^Ga]Ga-NODAGA-FAP647. In contrast, blocking effect for [^68^Ga]Ga-NODAGA-FAP800 was slightly lower (> − 80%). As shoulder joint uptake is associated with FAP expression in cartilage tissue, specifically chondrocytes, it can be speculated that both tracers bind murine to a similar extent as human FAP. Over 75% of uptake in the shoulder joints was blocked by pre-injection of unlabeled FAPI-04 as well.

Interestingly, uptake into target-negative WT-HT1080 xenografts was also reduced by − 55 to − 75% via an excess of unlabeled FAPI-04. While the total uptake into WT-HT1080 tumors was substantially lower compared to that of hFAP-HT1080, this effect is nevertheless noticeable. The growth of both HT1080 xenografts was relatively fast (reaching endpoints typically after 12–18 days), which reduces the chance of substantial invasion of murine tumor-associated fibroblasts. However, murine FAP could still account for some binding of both tracers in the WT-HT1080 tumors. Based on the temporal pattern of uptake, a sole blood-flow effect seems unlikely. This is further corroborated by the muscle uptake pattern, which is consistently lower, albeit not by much, than that of WT-HT1080 xenografts. Based on autoradiographic and fluorescence imaging of ex vivo tumor sections, WT-HT1080 tissue does not show any particular accumulation at the tumor borders (Supplementary Fig. 19, lower panel), which could indicate delayed infiltration. A rather homogenous distribution is observed, pointing to either a low but intrinsic expression, or a low-grade infiltration by murine fibroblasts immediately after injection.

Furthermore, pre-injection of unlabeled FAPI-04 apparently increased renal clearance of [^68^Ga]Ga-NODAGA-FAP647, based on radioactivity in the kidney compared to tracer-only injected animals. The increase was between 30 and 100%, depending on the timepoint and was not observed for [^68^Ga]Ga-NODAGA-FAP800.

### In vivo fluorescence imaging

**Fig. 6:**
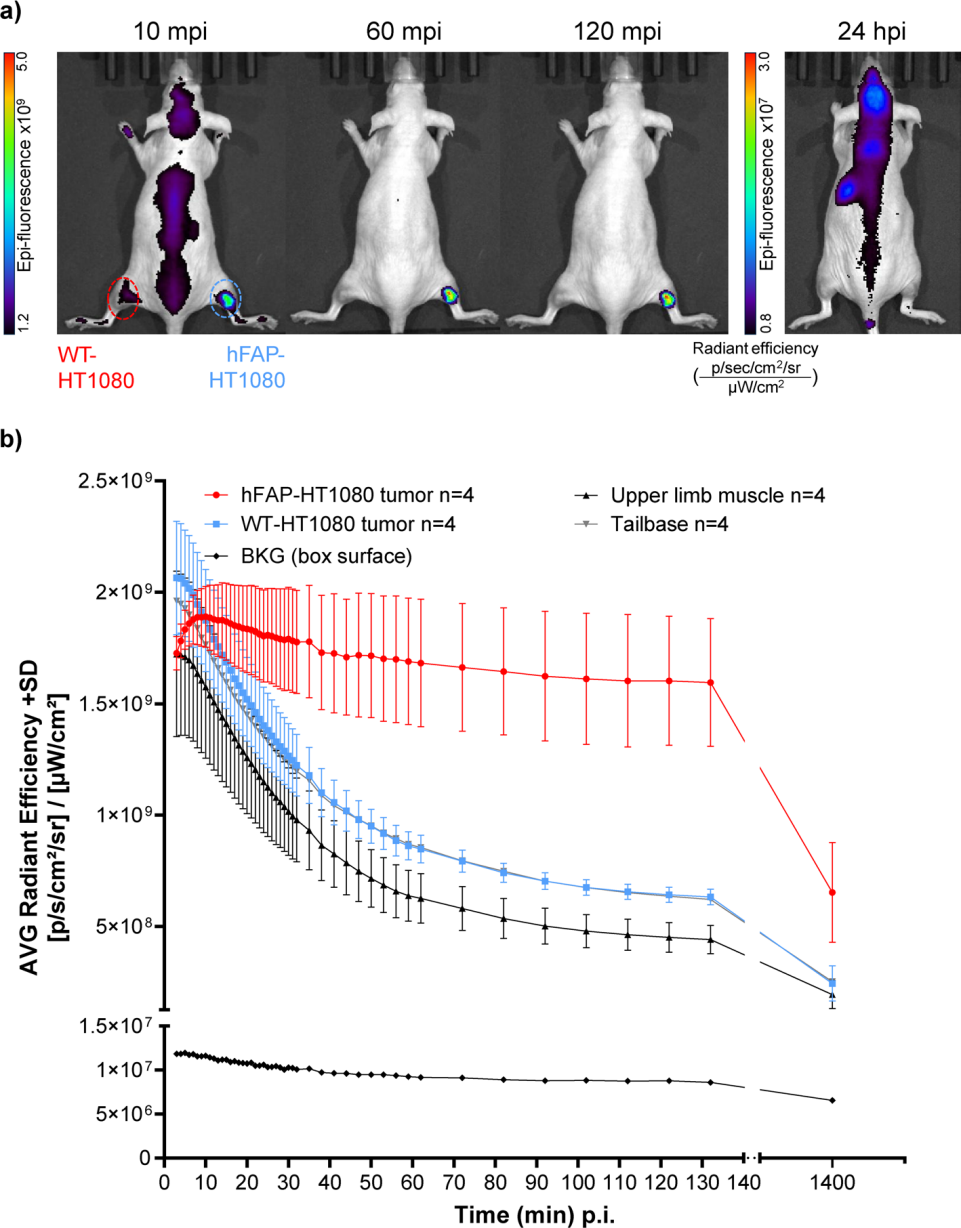
**a** Representative fluorescence images (from radiant efficiency, [p/s/cm^2^/sr] / [µW/cm^2^]) of NODAGA-FAP647 in nude mice carrying hFAP/WT-HT1080 xenografts (right/left thigh): Animals were injected with 0.09 mg/kg (*n* = 1) or 0.33 mg/kg (*n* = 3) and imaged 2–30 min post injection (mpi; every minute), 30–60 mpi (every 5 min) and 60–120 mpi (every 10 min). An additional image was taken at ~ 24 h post injection (hpi). Scale differs between 10/60/120 mpi and 24 hpi. **b** Fluorescence intensity (average radiant efficiency) over time and regions of interest (ROI) of all animals (*n* = 4)

In vivo fluorescence imaging of NODAGA-FAP647 again showed a fast blood flow-driven distribution of fluorescence (Fig. 6a), when quantified in all ROIs (tumors, muscle and tail base). All non-target ROIs showed a consistent decrease in fluorescence intensity over the first 120 min (Fig. 6b). In contrast, the hFAP-HT1080 tumor revealed a steady increase in signal over the first minutes, reaching a plateau at ~ 10 min post injection. This was followed by a slow decrease in signal intensity (Fig. 6b). When analyzing fluorescence intensity in hFAP-HT1080 and WT-HT1080 tumors, a two-way ANOVA with time and tumor entity as factors revealed a significant main effect of time [F (1.369, 8.378) = 22.74; *P* = 0.0007] but not tumor entity. However, a significant interaction of tumor entity and time was found [F (47, 282) = 8.088; *P* < 0.0001]. After 24 h, fluorescence in the target-positive tumor was still roughly 60% higher compared to the target-negative WT-HT1080 tumor or muscle. The ratio of target-positive to negative tumor (hFAP/WT-HT1080) and hFAP-HT1080/muscle demonstrated the target-specific accumulation of NODAGA-FAP647, with values (2.5 and 3.5 at 120 min post injection) being in a similar range as those observed in PET imaging (Supplementary Fig. 17).

Again, specificity of the compound was supported by the WT-HT1080 to muscle ratio (Supplementary Fig. 17, left-side graph), which was close to one over the imaging period of 24 h. Overall, this data implies that NODAGA-FAP647 offers a good temporal window for fluorescent imaging after injection.

**Fig. 7:**
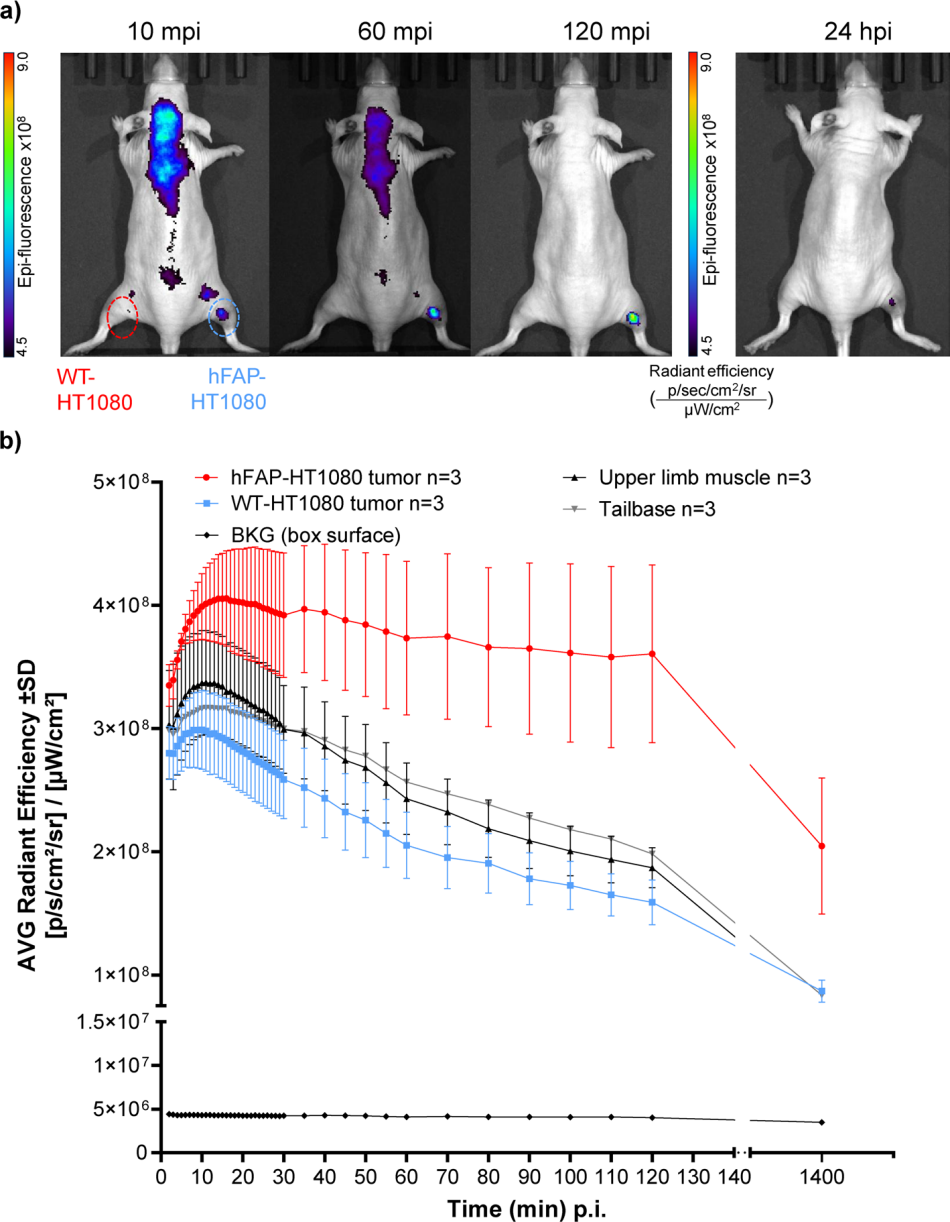
**a** Representative fluorescence images (from radiant efficiency, [p/s/cm^2^/sr] / [µW/cm^2^]) of NODAGA-FAP800 in nude mice carrying hFAP/WT-HT1080 xenografts (right/left thigh): Animals were injected with 0.17 mg/kg (*n* = 3) and imaged 2–30 min post injection (mp; every minute), 30–60 mpi (every 5 min) and 60–120 mpi (every 10 min). An additional image was taken at ~ 24 h post injection (hpi). **b** Fluorescence intensity (average radiant efficiency) over time and regions of interest (ROI) of all animals (*n* = 2)

A similar behavior was observed for NODAGA-FAP800 (Fig. 7a). Overall, the blood flow-driven distribution of the fluorescent signal appeared slower. Similarly, in all non-target ROIs, the decrease in signal intensity also appeared slower. Accumulation in hFAP-HT1080 xenografts supports this observation, plateauing at ~ 20 min post injection. There, in contrast to non-target regions, only a minimal decrease in signal was seen over the next 120 min (Fig. 7b). A two-way ANOVA with time and tumor entity as factors revealed a significant main effect of time [F (1.316, 5.264) = 10.09; *P* = 0.0198] and a near significant main effect of tumor entity [F (1, 4) = 6.374; *P* = 0.0650]. Again, a significant interaction of tumor entity and time [F (41, 164) = 2.168; *P* = 0.0003] was observed. As seen with NODAGA-FAP647, a robust signal advantage (60% higher) in the hFAP-HT1080 xenografts over WT-HT1080 or muscle tissue was found 24 hpi (Fig. 7a) and was qualitatively observed even beyond 24 hpi (Supplementary Fig. 18). The ratio between target-positive and negative tumor (~ 2.2 at 120 min post-injection) and tumor to muscle (~ 1.9 at 120 min post-injection) was somewhat lower than that observed for NODAGA-FAP647 (Supplementary Fig. 17, right-side graph). Similar to NODAGA-FAP647, the WT-HT1080 to muscle ratio, was ~ 1 over the imaging period, only increasing at 24 h post-injection. Overall, the data indicates that NODAGA-FAP800 accumulates slightly slower than NODAGA-FAP647, which can be explained to a certain extent by its overall slower binding kinetics observed in vitro.

## Discussion

Intraoperative fluorescence guidance has shown to be a cost-effective and sensitive technique, which can reduce the reoccurrence of cancer after surgery by up to 70% (Hernot et al. 2019; Tilly et al. 2014; Wong et al. 2012; Esposito et al. 2008; Vos et al. 2017). Cancer-specific probes offer advantages in specificity and sensitivity, as shown before for fluorescently-labeled antibodies or small molecules (Jongh et al. 2020; Voskuil et al. 2020). In the past decade, several multifunctional probes for bimodal imaging have been developed and examined, to be utilized for preoperative diagnostic noninvasive molecular imaging by PET and intraoperative fluorescence guidance (Kubeil et al. 2022; Minges et al. 2025). The utilization of a single molecule for both imaging techniques enables the precise co-localization of tumor lesions prior and during surgical resection of tumor tissue, due to the identical pharmacodynamics and kinetics for both modalities (Louie 2010; Minges et al. 2025). We sought to develop a multifunctional probe targeting FAP, a prominent biomarker for a broad variety of cancer entities (Fitzgerald and Weiner 2020).

The small molecule inhibitor UAMC1110 was chosen as lead structure and combined with the linker of the radiotracer FAPI-46, which is already clinically applied and displays favorable pharmacokinetic properties (Jansen et al. 2014; Loktev et al. 2019). The two cyanine dyes AlexaFluor647 and IRdye800CW were selected for the conjugation, to cover an emission wavelength from the far-red to the near-infrared region. Furthermore, several conjugates of the IRDye800CW dye are currently under investigation in clinical phase I/II trials. (NCT03510208, NCT03384238, NCT04511078, NCT04511078).

The binding capacity and affinity towards FAP of the synthesized inhibitors was assessed by different assays and FAP-expressing specimens, along with selectivity over DPPIV. The latter was found to be similarly high as reported for other FAP radioligands (Köchel et al. 2025; Kochel et al. 2025; Tanc et al. 2024; Adhikari et al. 2025; Poulie et al. 2023). Importantly, a fluorogenic enzyme inhibition assay showed that both compounds retained the inhibitory activity towards FAP. Picomolar inhibition constants (*K*_i_) indicated a tight binding profile. Tight binding inhibitors form a highly stable inhibitor-enzyme complex, typically associated with a slow dissociation process and low values for the dissociation rate constant *k*_d_ (Copeland 2013; Williams and Morrison 1979). Using a real-time binding assay, association *k*_a_ and dissociation rate constants *k*_d_ were determined separately. Here, a low *k*_d_ and therefore a very slow dissociation of both compounds was observed. To rule out internalization of the ligands, different FAP-expressing specimens were used: (1) tissue sections from frozen hFAP-HT1080 xenografts and (2) PFA-fixed cells. Both conditions either preclude active transport processes into the cell (PFA-fixed cells) or are characterized by an absence of cell-membrane integrity (frozen tissue sections). Comparable *k*_d_ values were determined on those FAP-expressing specimens compared to live hFAP-HT0180 cells. Furthermore, an acid wash did not result in any discernible loss of signal, again supporting slow dissociation instead of internalization.

Generally, nitrile-based covalent interactions with serine of cysteine residues are reversible, with observed residence times of minutes to several hours for structurally similar serine protease inhibitors (Tummino and Copeland 2008). However, association and dissociation rates can be influenced by secondary interactions of the ligand in the binding pocket (Bonatto et al. 2023). A study by Van Rymenant et al. (Van Rymenant et al. 2021) with Cy3/5 conjugates of the FAP inhibitor UAMC1110 demonstrated a long-lasting, practically irreversible inhibition of the enzymatic activity of FAP over seven days. It was hypothesized by the authors that ionic interactions of the deprotonated sulfonic acid moieties in the cavity inside the protein could be responsible for the formation of a stable inhibitor-enzyme complex. A similar mechanism could explain the behavior of our compounds.

In vivo PET demonstrated a rapid biodistribution for both radiotracers. [^68^Ga]Ga-NODAGA-FAP647 and [^68^Ga]Ga-NODAGA-FAP800 showed a fast systemic distribution, rapid accumulation in the hFAP-HT1080 tumor and fast clearance from healthy tissue via the kidneys. As a result, a high imaging contrast was achieved from 10 min post injection onwards. The only apparent off-target accumulation was observed in the shoulder joints, shown to be mediated (in part) by FAP, as prior injection of unlabeled FAPI-04 attenuated this uptake by over 75%. In vivo fluorescence imaging showed comparable kinetic behavior as in PET imaging for NODAGA-FAP647 and NODAGA-FAP800. Uptake in the hFAP-HT1080 tumor increased steadily up to 10 min post injection, showing a long tumor retention afterwards. Clearance from healthy tissue occurred rapidly as previously seen in PET. After 24 h post injection, the fluorescence signal was only reduced by 59% and 43% for NODAGA-FAP647 and NODAGA-FAP800, respectively, reflecting the slow dissociation which was determined in vitro. The fast clearance time from healthy tissue and long tumor retention are favorable properties for the clinical application, as a high contrast and broad temporal window from the application to the intra-operative detection is achieved.

So far, a series of conjugates of FAP inhibitors with fluorescent dyes for in vivo optical imaging have been reported in the literature (Van Rymenant et al. 2021; Heide et al. 2024; Mukkamala et al. 2022; Chen et al. 2025, 2024; Dvorakova et al. 2017; Zettlitz et al. 2018; Rizzo et al. 2025; Narain et al. 2024; Roy et al. 2020). However, to the best of our knowledge, only two bimodal FAP-targeting radiotracers for optical and PET imaging have been reported in the literature. Zhang et al. (Zhang et al. 2024) reported a conjugate derived from the radiotracer FAPI-04 conjugated with the fluorescent dye fluorescein named FAPI-FAM. FAPI-FAM was conjugated with the chelator DOTA for radiolabeling with gallium-68. The compound exhibited an IC_50_ value in the low nanomolar range for FAP. Further, in vivo fluorescence imaging in mice demonstrated a high tumor retention for FAPI-FAM, with a decrease of signal intensity of 25% after twenty-four hours. Zhao et al. (Zhao et al. 2024) reported a NOTA-bearing ligand for radiolabeling with aluminum-[^18^F]fluoride named NOTA-FAPI-MB. The structure was also based on FAPI-04 linked to the fluorescent dye methylene blue (MB). The authors also reported a high in vivo tumor retention, with a 54% decrease of signal intensity after twenty-four hours. The prolonged retention time was attributed to interactions of the positively charged dye MB to anionic nucleic acids. FAPI-FAM and NOTA-FAPI-MB showed comparable tumor retention times to our presented derivatives. Additionally, it implies that the introduction of an ionic dye likely creates secondary interactions with the target, as we have also hypothesized for NODAGA-FAP647/800. However, the binding kinetics of FAPI-FAM and NOTA-FAPI-MB were not investigated. An in-detail investigation of the kinetic binding parameters could provide a better understanding of the inhibitor-enzyme interactions and can help to estimate the in vivo performance and therefore rationally develop such ligands.

In summary, the two presented ligands show suitable pharmacokinetic properties and tumor uptake for a first in-human application. In the future, it may be advantageous to substitute the employed dyes with those of higher photostability (Bandi et al. 2022) and a further shift into the short-wave infrared range to benefit tissue penetration and resolution. This would further improve applicability in fluorescence-guided surgery.

## Conclusion

Two FAP-targeting ligands for PET imaging and optical fluorescence imaging in the NIR and the far-red region were synthesized and preclinically evaluated in vitro and in vivo. Small animal PET and optical fluorescence imaging showed rapid biodistribution and selective accumulation in FAP-expressing tumors, resulting in high image contrast. The comparably long tumor retention is especially advantageous for the application as fluorescent probe in intraoperative fluorescence-guided surgery. Both of our FAP-targeting multifunctional ligands for bimodal imaging represent suitable candidates for first in-human application and translation into the clinic.

## Materials and methods

### Organic synthesis

#### General

All chemicals were purchased from Sigma Aldrich (München Germany), BLDpharm (Shanghai, China), Thermo Fisher Scientific (Darmstadt, Germany), VWR (Bruchsal, Germany,) ABCR (Karlsruhe, Germany), Acros Organics (Geel, Belgium) or Carl Roth (Karlsruhe, Germany) at highest purity grades available. TLC analysis was performed on ChromaPlate Alu 60 UV silica gel TLC plates (Carl Roth, Karlsruhe, Germany) with fluorescent indicator and visually analyzed at 254 nm. Automated flash chromatography was performed on a Selekt System from Biotage (Uppsala, Sweden) with peak detection at 254 nm and 300 nm. ESI–MS analysis was performed using an AQUITY UPLC I-class plus system coupled to a XEVO TQ-S mass spectrometer from Waters (Milford, Massachusetts, USA). Analytical HPLC analysis was performed on a modular LC20A system from Shimadzu (Kyoto, Japan), consisting of a DGU-20A 5R degasser, two LC-20AR pumps, a CTO-20AC column oven with column switching valve, a SIL-20AC HT autosampler for analytical samples, a SPD-M20A diode array detector, a FRC-10A fraction collector and a CBM-20A communication bus module. All analytical and preparative samples were separated on column at 50 °C. NMR analysis was performed on an Agilent Technologies (Santa Clara, CA, USA) 400 MR spectrometer, consisting of a 400/54 compact magnet, a 400 MR console and a 400 MHz OneNMR probe. Chemical shifts δ are reported in ppm and referenced to the residual solvent signal for ^1^H and ^13^C spectra and to CFCl_3_ (δ_F_ = 0.00 ppm) for ^19^F spectra. All spectra were analyzed using the software MestreNova version 15.0.1–35,756. High resolution mass spectrometry was performed on an Agilent 6575 Revident LC/Q-TOF system with electron spray ionization (ESI). Compound **6** was synthesized following the procedure from Jansen et al. (Jansen et al. 2014) Intermediate **8** was synthesized by an adapted synthesis from Loktev et al. (Loktev et al. 2019) as shown in the reaction scheme in Supplementary Fig. 1. FAPI-04 was synthesized by a similar procedure from Lindner et al. (Lindner et al. 2018).

#### Analytical HPLC

Analytical HPLC analysis was performed with a mixture of water + 0.1% TFA (A) and acetonitrile + 0.1% TFA (B) as mobile phase using a Jupiter Proteo C-18, 250 × 4.6 mm (Phenomenex, Torrance, CA, USA) and a flow of 1.0 mL/min. The following method was utilized (A/B):

$$ \begin{aligned} t_{{0{\text{ min}}}} & \, = {95}/0{5}{-}t_{{\text{5 min}}} = {95}/0{5}{-}t_{{{3}0 {\text{min}}}} = 0{5}/{95}{-}t_{{\text{34 min}}} \\ & \, = 0{5}/{95}{-}t_{{\text{37 min}}} = {95}/0{5}{-}t_{{\text{42 min}}} = {95}/0{5} \\ \end{aligned} $$

Analytical HPLC chromatograms of NODAGA-FAP647 and NODAGA-FAP800 are provided in the supplementary information. (Supplementary Fig. 3, 5).

#### LC–MS

The following gradients were used for LC–MS analysis with water + 0.1% acetic acid (C) and acetonitrile + 0.1% acetic acid (D) as mobile phase, a flow of 0.4 mL/min on an Acquity BEH C18 column from Agilent (2.1 mm × 100 mm, 1.7 μM). The following methods were utilized (C/D):

$$ \begin{gathered} {\text{method1}}:t_{{0{\text{min}}}} = {95}/0{5} - t_{{0.{\text{5min}}}} = {95}/0{5}{-}t_{{{5}.{\text{5min}}}} \hfill \\ \quad \quad \quad \quad = 0{5}/{95}{-}t_{{{7}.0{\text{min}}}} = 0{5}/{95}{-}t_{{{8}.0{\text{min}}}} = {95}/0{5}{-}t_{{{8}.{\text{5min}}}} = {95}/0{5}, \hfill \\ {\text{method2}}:t_{{0{\text{min}}}} = {95}/0{5} - t_{{0.{\text{5min}}}} = {95}/0{5}{-}t_{{{5}.{\text{5min}}}} = {45}/{55} - t_{{{6}.0{\text{min}}}} \hfill \\ \quad \quad \quad \quad = 0{5}/{95}{-}t_{{{7}.0{\text{min}}}} = 0{5}/{95}{-}t_{{{8}.0{\text{min}}}} = {95}/0{5}{-}t_{{{8}.{\text{5min}}}} = {95}/0{5}, \hfill \\ {\text{method3}}:t_{{0{\text{min}}}} = {55}/{45} - t_{{0.{\text{5min}}}} = {55}/{45}{-}t_{{{5}.{\text{5min}}}} = 0{5}/{95} - t_{{{6}.0{\text{min}}}} \hfill \\ \quad \quad \quad \quad = 0{5}/{95}{-}t_{{{7}.0{\text{min}}}} = 0{5}/{95}{-}t_{{{8}.0{\text{min}}}} = {55}/{45}{-}t_{{{8}.{\text{5min}}}} = {55}/{45} \hfill \\ \end{gathered} $$

#### Synthetic procedures


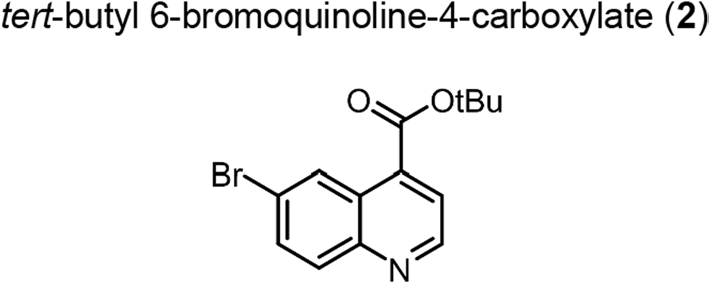


6-Bromo-4-carboxylic acid (**1**) (9.921 g, 39.36 mmol) and *N*,*N*-Dimethylpyridin-4-amine (2.406 g, 19.69 mmol) was placed in a Schlenk flask, which was evacuated and filled with an argon atmosphere three times. THF (anhydrous, 50.0 mL) was added resulting in a dark brown suspension. Di-*tert*-butyl dicarbonate (45.21 mL, 196.8 mmol) was added while cooling to 0 °C. Subsequently the reaction was refluxed at 75 °C for 16 h. After cooling to room temperature, the solvent was evaporated under reduced pressure and the residue was dissolved in ethyl acetate, washed three times with saturated bicarbonate solution and brine, dried over sodium sulfate, filtered and the solvent evaporated under reduced pressure. The crude was purified by column chromatography on silica (ethyl acetate/ n-hexane: 1:2). The product was obtained as yellow solid (11.36 g, 36.86 mmol, 93%). TLC: R_f_ = 0.67 (ethyl acetate/ n-hexane, 1:2), LC–MS (LC method 1, ESI^+^): *t*_R_ = 4.53 min, calculated for C_14_H_14_BrNO_2_: m/z = 308.03 [M + H]^+^, found m/z = 308.18 [M + H]^+^, ^1^H NMR (400 MHz, CDCl_3_) δ 9.02 (d, *J* = 2.2 Hz, 1H), 8.99 (d, *J* = 4.4 Hz, 1H), 8.01 (d, *J* = 9.0 Hz, 1H), 7.87 (d, *J* = 4.4 Hz, 1H), 7.82 (dd, *J* = 9.0, 2.2 Hz, 1H), 1.68 (s, 9H).^, 13^C NMR (101 MHz, CDCl_3_) δ 165.01, 150.30, 147.90, 135.71, 133.28, 131.74, 128.31, 126.44, 122.98, 122.71, 83.49, 28.36.


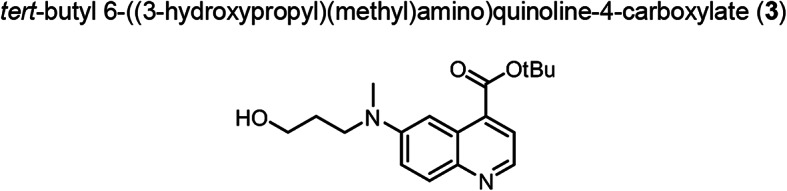


Compound (**2**) (1.527 g, 4.955 mmol), (Methylamino)propan-1-ol (1.394 g, 15.64 mmol) and Cs_2_CO_3_ (8.037 g, 24.67 mmol) were placed in a Schlenk flask under argon atmosphere. Toluene (anhydrous, 150.0 mL) was added resulting in a yellow suspension, which was degassed by passing an argon stream through the suspension. Tris(dibenzylideneacetone)dipalladium (0.158 g, 0.172 mmol) and BINAP (0.202 g, 0.325 mmol) were added to the mixture under argon stream. The reaction mixture was stirred at 60 °C for 43 h. After cooling to room temperature, the solvent was evaporated under reduced pressure and the residue was dissolved in a mixture of ethyl acetate and dichloromethane and filtered over celite. The solvent of the filtrate was removed, the residue partitioned between dichloromethane and brine. The phases were separated and the aqueous phase extracted with dichloromethane two times. The combined organic phases were dried over sodium sulfate, filtered and the solvent evaporated under reduced pressure. The crude was purified by flash chromatography on silica (methanol/ dichloromethane, gradient: 2 → 10%). The product was obtained as red oil (0.863 g, 2.727 mmol, 55%). TLC: R_f_ = 0.46 (dichloromethane/ methanol, 9:1), LC–MS (LC method 1, ESI^+^): *t*_R_ = 4.50 min, calculated for C_18_H_24_N_2_O_3_: m/z = 317.19 [M + H]^+^, found m/z = 317.26 [M + H]^+^, ^1^H NMR (400 MHz, CDCl_3_) δ 8.62 (d, *J* = 4.5 Hz, 1H), 7.96 (d, *J* = 9.4 Hz, 1H), 7.82 (d, *J* = 2.8 Hz, 1H), 7.73 (d, *J* = 4.5 Hz, 1H), 7.41 (dd, *J* = 9.4, 2.9 Hz, 1H), 5.29 (s, 1H), 3.76 (t, *J* = 5.9 Hz, 2H), 3.64 (t, *J* = 6.9 Hz, 2H), 3.11 (s, 3H), 1.95 – 1.86 (m, 2H), 1.66 (s, 9H). ^13^C NMR (101 MHz, CDCl_3_) δ 166.25, 148.76, 144.03, 142.96, 133.91, 130.25, 127.46, 122.81, 119.55, 102.47, 82.52, 60.25, 49.55, 38.74, 30.12, 28.40.


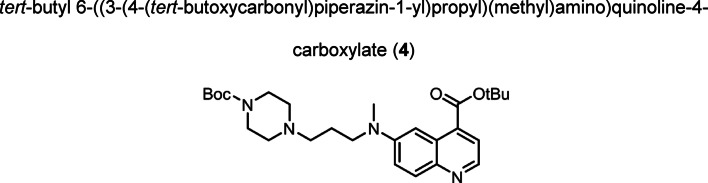


Compound (**3**) (0.842 g, 2.661 mmol) was dissolved in dichloromethane (anhydrous, 20.0 mL) and DIPEA (2.500 mL, 14.35 mmol) was added to the solution. Mesyl chloride (0.650 mL, 8.399 mmol) was added dropwise over 5 min while cooling to 0 °C. The reaction was allowed to warm to room temperature and stirred for 2 h. The solvent was removed under reduced pressure. The residue was dissolved in DMF (anhydrous, 20.0 mL). *N*-Boc-piperazine (1.632 g, 8.762 mmol), potassium iodide (0.287 g, 1.729 mmol) and DIPEA (2.500 mL, 14.35 mmol) were added to the solution and the reaction was stirred at 80 °C for 18 h. The solvent was removed, the residue was dissolved in ethyl acetate and washed with saturated sodium bicarbonate solution. The aqueous phase was extracted with ethyl acetate. The combined organic phases were dried over sodium sulfate, filtered and the solvent was evaporated. The crude product was purified by column chromatography (cyclohexane/ acetone, 5:1 → 1:1 + 1% triethylamine). The product was obtained as red oil (0.702 g, 1.448 mmol, 54%). TLC: R_f_ = 0.56 (cyclohexane/ acetone, 1:1 + 1% triethylamine), LC–MS (LC method 1, ESI^+^): *t*_R_ = 3.97 min, calculated for C_27_H_40_N_4_O_4_: m/z = 485.31 [M + H]^+^, found m/z = 484.93 [M + H]^+^, ^1^H NMR (400 MHz, CDCl_3_) δ 8.62 (d, *J* = 4.5 Hz, 1H), 7.94 (d, *J* = 9.4 Hz, 1H), 7.79 (d, *J* = 2.8 Hz, 1H), 7.73 (d, *J* = 4.5 Hz, 1H), 7.43 (dd, *J* = 9.4, 2.9 Hz, 1H), 3.55 (t, *J* = 7.0 Hz, 2H), 3.42 (dt, *J* = 13.9, 5.1 Hz, 4H), 3.09 (s, 3H), 2.44 – 2.32 (m, 6H), 1.87 – 1.78 (m, 2H), 1.66 (s, 9H), 1.45 (s, 9H). ^13^C NMR (101 MHz, CDCl_3_) δ 166.39, 154.89, 148.53, 144.87, 143.78, 133.33, 130.81, 127.38, 122.86, 119.18, 102.43, 82.15, 79.74, 55.63, 53.14, 53.03, 50.56, 38.70, 31.05, 28.56, 28.44.


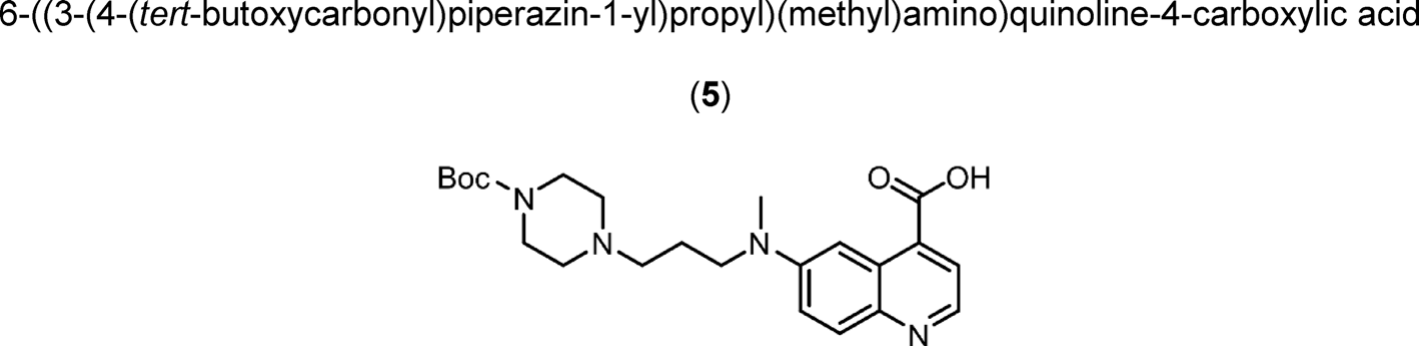


Compound (**4**) (0.687 g, 1.420 mmol) was dissolved in DMF (20.0 mL) forming a red solution. Sodium hydride (60% w/w in mineral oil, 0.458 g, 11.45 mmol) was added portion-wise over 10 min. Afterwards, the resulting suspension was stirred at room temperature for 18 h. Reaction control by TLC showed remaining starting material. Additional sodium hydride (60% w/w in mineral oil, 0.339 g, 8.475 mmol) was added portion-wise and the reaction was stirred at room temperature for 24 h. After completion, the solvent was removed under reduced pressure. The crude product was purified by flash chromatography on silica (acetonitrile/ ethanol: 5 → 65% + 1% triethylamine) The product was obtained as orange solid (0.411 g, 0.959 mmol, 68%). TLC: R_f_ = 0.25 (C-18 silica, water/ acetonitrile, 50:50 + 0.1% trifluoroacetic acid), LC–MS (LC method 1, ESI^+^): *t*_R_ = 2.24 min, calculated for C_23_H_32_N_4_O_4_: m/z = 429.25 [M + H]^+^, found m/z = 429.24 [M + H]^+^, ^1^H NMR (400 MHz, CD_3_CN) δ 8.60 (d, *J* = 5.0 Hz, 1H), 8.02 (d, *J* = 9.4 Hz, 1H), 7.93 (d, *J* = 5.0 Hz, 1H), 7.75 (s, 1H), 7.54 (dd, *J* = 9.4, 2.0 Hz, 1H), 4.91 – 2.57 (m, 4H), 3.54 (t, *J* = 7.2 Hz, 2H), 3.22 – 3.10 (m, 2H), 3.06 (s, 3H), 2.16 – 2.04 (m, 2H), 1.43 (s, 9H). ^13^C NMR (101 MHz, CDCl_3_) δ 188.17*, 172.28, 154.16, 145.70, 145.48, 142.74, 133.10, 130.79, 128.12, 126.80, 107.62, 85.53, 59.38, 56.68, 54.52, 45.72 (br s), 43.21, 26.53. *peak determined from HMBC spectrum.


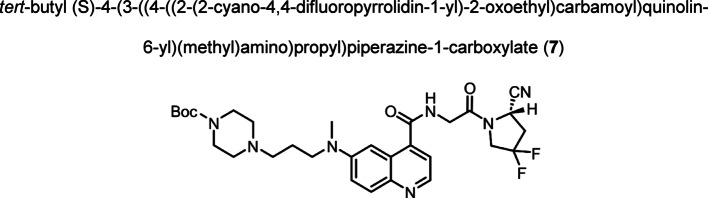


Compound (**5**) (0.386 g, 0.901 mmol) and HATU (0.997 g, 2.622 mmol) were dissolved in DMF (anhydrous, 25.0 mL) and DIPEA (1.5 mL, 8.610 mmol) was added. The mixture was stirred for 15 min at room temperature. A solution of compound (**6**) (0.401 g, 1.777 mmol) in DMF (anhydrous, 5.0 mL) was added dropwise and the reaction was stirred at room temperature for 19 h. The solvent was removed under reduced pressure, the residue dissolved in ethyl acetate, and washed with saturated sodium bicarbonate solution. The aqueous phase was extracted with ethyl acetate. The combined organic phases were washed with brine, dried over sodium sulfate, filtered and the solvent was evaporated. The crude product was purified by reverse phase flash chromatography on C-18 silica (2 → 30% MeCN in H_2_O + 0.1% trifluoroacetic acid). The product was obtained as yellow solid (0.617 g, 1.029 mmol, 75%). TLC: R_f_ = 0.37 (dichloromethane/ methanol, 9:1 + aq. ammonia solution), LC–MS (LC method 1, ESI^+^): *t*_R_ = 2.53 min, calculated for C_30_H_39_F_2_N_7_O_4_: m/z = 600.31 [M + H]^+^, found m/z = 600.14 [M + H]^+^, ^1^H NMR (400 MHz, CD_3_CN) δ 8.64 (d, *J* = 5.1 Hz, 1H), 8.16 (d, *J* = 9.5 Hz, 1H), 8.03 (t, *J* = 5.4 Hz, 1H), 7.67 – 7.61 (m, 2H), 7.56 (d, *J* = 2.3 Hz, 1H), 5.07 (dd, *J* = 9.2, 2.8 Hz, 1H), 4.38 – 3.93 (m, 4H), 3.77 – 3.54 (m, 2H), 4.84 – 2.59 (m, 8H), 3.18 – 3.10 (m, 3H), 2.97 – 2.70 (m, 1H), 2.18 – 2.03 (m, 2H), 1.42 (s, 9H). ^13^C NMR (101 MHz, CD_3_CN) δ 183.61, 168.92, 167.46, 150.02, 140.25, 140.01, 135.54, 128.56, 127.45 (t,* J* = 248.4 Hz), 124.62, 123.89, 120.76, 118.44, 102.78, 81.16, 54.93, 52.74 (t,* J* = 32.2 Hz), 52.15, 49.85, 45.56, 45.50, 42.79, 41.3 (br s), 39.04, 37.72 (t, *J* = 25.1 Hz), 28.35, 21.89. ^19^F NMR (376 MHz, CD_3_CN) δ −94.09 to -96.91 (m), −103.09 to −107.05 (m).

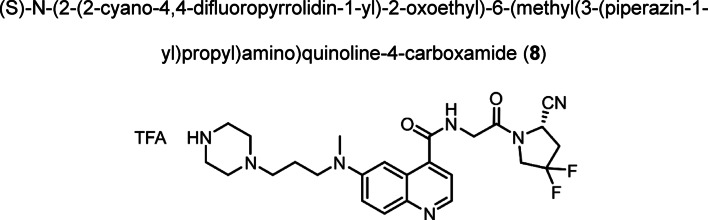



Compound (**7**) (0.302 g, 0.504 mmol) was dissolved in DCM (anhydrous, 4.0 mL) and TIPS (1.0 mL, 10% v/v) was added. TFA (5.0 mL, 50% v/v) was added while cooling to 0 °C. The reaction was allowed to warm to room temperature and stirred for 45 min. The solvent was removed under reduced pressure and further dried under high vacuum. The product was obtained as red oil and used directly without further purification. A small fraction of the product was purified by reverse phase flash chromatography on C-18 silica (2 → 25% MeCN in H_2_O + 0.1% trifluoroacetic acid) for NMR analysis. TLC: R_f_ = 0.06 (dichloromethane/ methanol, 9:1 + aq. ammonia solution), LC–MS (LC method 1, ESI^+^): *t*_R_ = 1.96 min, calculated for C_25_H_31_F_2_N_7_O_2_: m/z = 500.26 [M + H]^+^, found m/z = 500.28 [M + H]^+^_,_^1^H NMR (400 MHz, CD_3_CN) δ 8.65 (d, *J* = 5.0 Hz, 1H), 8.14 (d, *J* = 9.5 Hz, 1H), 7.99 – 7.92 (m, 1H), 7.62 (dd, *J* = 9.5, 2.8 Hz, 1H), 7.59 (d, *J* = 5.0 Hz, 1H), 7.56 (d, *J* = 2.8 Hz, 1H), 5.09 (dd, *J* = 9.2, 3.0 Hz, 1H), 4.31 – 3.95 (m, 6H), 3.72 – 3.57 (m, 2H), 3.27 (s, 3H), 3.98 – 2.65 (m, 8H), 3.16 – 3.05 (m, 26H), 2.95 – 2.72 (m, 2H), 2.15 – 2.01 (m, 2H). ^13^C NMR (101 MHz, CD_3_CN) δ 169.10, 167.97, 161.12 (q, *J* = 34.0 Hz), 149.91, 145.37, 141.46, 137.31, 128.36, 127.56 (t, *J* = 248.6 Hz), 123.22, 120.65, 119.40, 117.93 (q, *J* = 293.8 Hz), 102.92, 55.17, 52.78 (t, *J* = 32.3 Hz), 49.89, 49.82, 49.19, 45.63, 42.80, 41.91, 37.79 (t, *J* = 24.9 Hz), 22.15, 18.61, 17.35. ^19^F NMR (376 MHz, CD_3_CN) δ -74.85 (s), -95.46 to -96.67 (m), -103.29 to -104.37 (m).


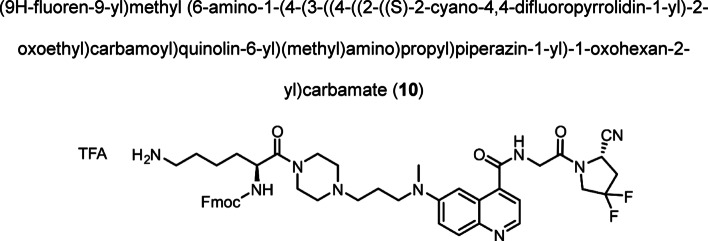


Fmoc-Lys(Boc)-OH **9** (0.309 g, 0.659 mmol), HATU (0.347 g, 0.913 mmol) and DIPEA (0.500 mL, 2.870 mmol) were dissolved in DMF (anhydrous, 2.0 mL). A solution of compound (**8**) (0.150 g, 0.300 mmol) in DMF (anhydrous, 1.5 mL) was added dropwise and the reaction mixture was stirred at room temperature for 1 h. After consumption of the starting material, the solvent was removed under reduced pressure. The residue was dissolved in DCM (anhydrous, 4.0 mL) and TIPS (1.0 mL, 10% v/v) was added. TFA (5.0 mL, 50% v/v) was added while cooling to 0 °C. Subsequently, the reaction was allowed to warm to room temperature. After 1.5 h of stirring the solvent was removed under reduced pressure. The crude product was purified by preparative HPLC. The product was obtained as red fluffy solid after lyophilization (0.198 g, 0.205 mmol, 68%). Analytical HPLC: *t*_R_ = 20.29 min, LC–MS (LC method 1, ESI^+^): *t*_R_ = 3.43 min, calculated for C_46_H_53_F_2_N_9_O_5_: m/z = 850.42 [M + H]^+^, found m/z = 850.34 [M + H]^+^, ^1^H NMR (400 MHz, DMSO-*d*_*6*_) δ 9.08 (t, *J* = 6.0 Hz, 1H), 8.68 (d, *J* = 4.5 Hz, 1H), 7.94 (d, *J* = 9.3 Hz, 1H), 7.89 (d, *J* = 7.5 Hz, 2H), 7.76 (s, 4H), 7.72 – 7.69 (m, 2H), 7.74 – 7.58 (m, 1H), 7.57 (d,, *J* = 9.1 Hz 1H), 7.49 (d, *J* = 4.4 Hz, 1H), 7.45 (d, *J* = 2.7 Hz, 1H), 7.42 (t, *J* = 7.4 Hz, 2H), 7.32 (t, *J* = 7.4 Hz, 2H), 5.68 (d, *J* = 8.8 Hz, 0.1H), 5.14 (dd, *J* = 9.2, 2.8 Hz, 0.9H), 4.51 – 3.81 (m, 10H), 3.69 – 3.43 (m, 6H), 3.32 – 3.02 (m, 2H), 3.05 (s, 3H), 3.01 – 2.68 (m, 4H), 2.05 – 1.92 (m, 2H), 1.64 – 1.38 (m, 4H), 1.43 – 1.20 (m, 2H). ^13^C NMR (101 MHz, DMSO-*d*_*6*_) δ 170.38, 168.01, 167.50, 158.35 (d, *J* = 32.8 Hz), 156.01, 147.61, 143.78, 143.74, 140.74, 129.32, 127.68, 127.06, 126.87 (t,* J* = 246.4 Hz), 126.27, 125.24, 124.38, 120.27, 120.15, 119.32, 102.44, 65.66, 51.23 (t, *J* = 31.7 Hz), 48.79, 46.64, 44.23 (d, *J* = 6.1 Hz), 41.37, 38.61, 38.19, 36.36 (t, *J* = 24.2 Hz), 26.74. ^19^F NMR (376 MHz, DMSO-*d*_*6*_) δ -73.57 (s), -93.03 to -96.04 (m), − 102.93 to -106.15 (m).


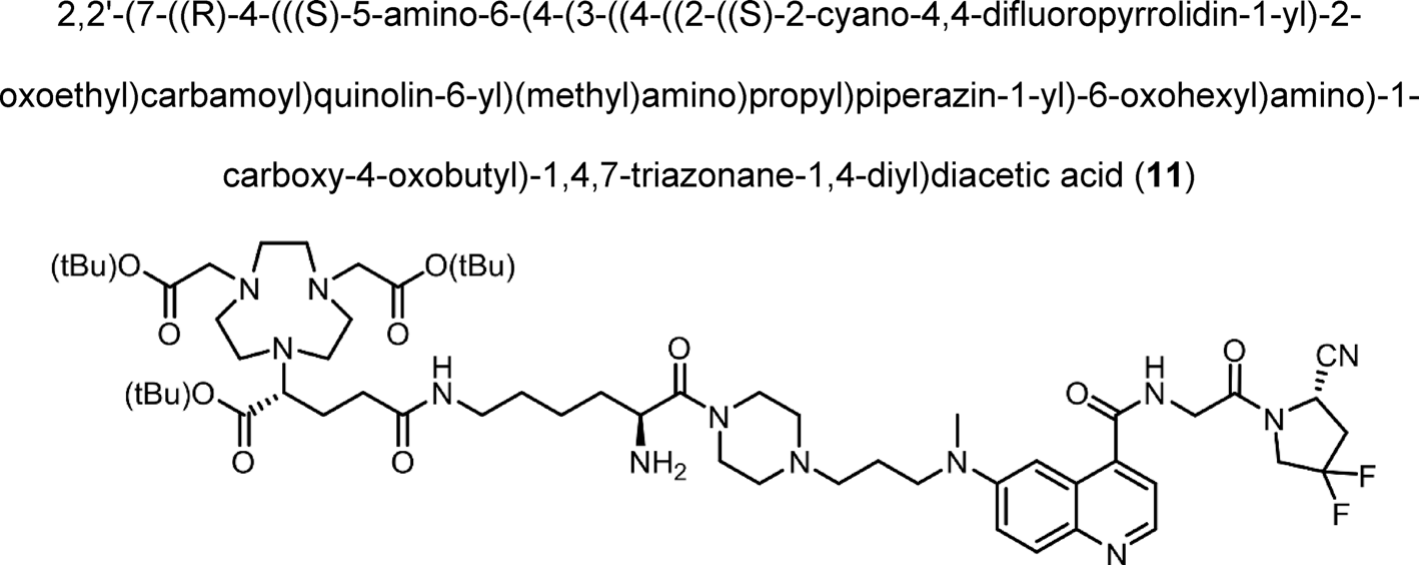


(R)-NODAGA-tris(*t*-Bu) ester (0.058 g, 0.106 mmol), HATU (0.076 g, 0.200 mmol) and DIPEA were dissolved in DMF (500 µL). A solution of compound (**10**) (0.082 g, 0.096 mmol) in DMF (800 µL) was added dropwise to the reaction and stirred for 1.5 h at room temperature. Piperidine (300 µL, 20% v/v) was added and the reaction stirred for another 1.5 h. The solvent was removed under reduced pressure and the crude product was purified by preparative HPLC. The product was obtained as red fluffy solid after lyophilization (0.078 g, 0.068 mmol, 80%). Analytical HPLC: *t*_R_ = 21.69 min, LC–MS (LC method 1, ESI^+^): t_R_ = 3.09 min, calculated for C_58_H_90_F_2_N_12_O_10_: m/z = 1153.69 [M + H]^+^, found m/z = 1153.59 [M + H]^+^, HRMS (ESI^+^-ToF): calculated for C_58_H_90_F_2_N_12_O_10_: m/z = 1153.6944 [M + 2H]^2+^, found m/z = 1153.6948 [M + 2H]^2+^.


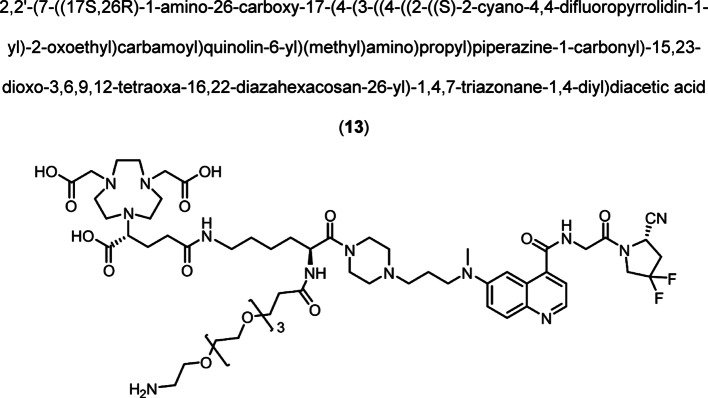


To a solution of amine (**11**) (10.40 mg, 5.81 µmol) and DIPEA (20 µL, 0.115 µmol) in DMF (750 µL), a solution of *N*-Boc-PEG_4_-propionic *N*-hydroxysuccinimidyl ester **12** (5.30 mg, 11.46 µmol) in of DMF (250 µL) was added dropwise and stirred for 2 h. The solvent was removed and the residue dissolved in of anhydrous DCM (anhydrous, 800 µL) and TIPS (200 µL, 20% v/v). TFA (1.0 mL, 50% v/v) was added dropwise while cooling to 0 °C. The reaction was to 40 °C and stirred for 2 h. The solvent was removed under reduced pressure and the crude product purified by preparative HPLC. The product was obtained as red fluffy solid after purification. (4.22 mg, 4.43 µmol,59%). Analytical HPLC: *t*_R_ = 15.82 min, LC–MS (method 1, ESI^+^): *t*_R_ = 2.06 min, calculated for C_57_H_87_F_2_N_13_O_15_: m/z = 1232.65 [M + H]^+^, found m/z = 1232.45 [M + H]^+^, HRMS (ESI^+^-ToF): calculated for C_57_H_87_F_2_N_13_O_15_: m/z = 616.8279 [M + 2H]^2+^, found m/z = 616.8277 [M + 2H]^2+^.


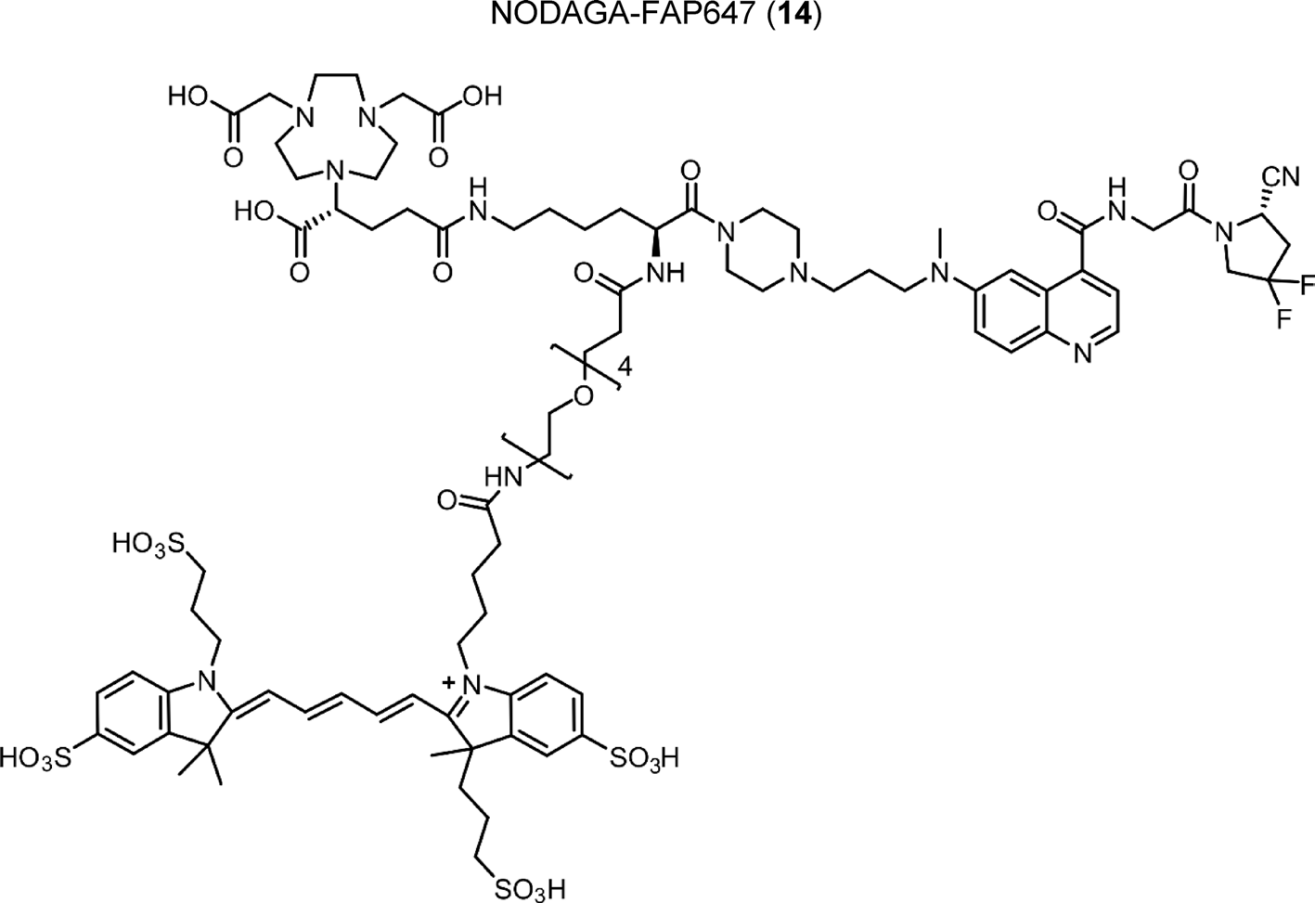


To a solution of AlexaFluor647-NHS ester (1.0 mg, 0.785 µmol) and DIPEA (5.0 µL, 28.70 µmol) in of DMF (anhydrous, 800 µL) a solution of compound (**13**) (1.34 mg, 1.087 µmol) in DMF (200 µL) was added and the reaction stirred at room temperature for 2 h. The solvent was removed under reduced pressure. The crude product was purified by preparative HPLC. The product was obtained as dark blue fluffy solid after lyophilization (1.27 mg, 0.612 µmol, 78%). Analytical HPLC: *t*_R_ = 16.72 min, HRMS (ESI^−^-ToF): calculated for C_94_H_133_F_2_N_15_O_28_S_4_: m/z = 1028.3955 [M-2H]^2−^, found m/z = 1028.3929 [M-2H]^2−^ The original HRMS spectrum is provided in the supplementary information. (Supplementary Fig. 4).


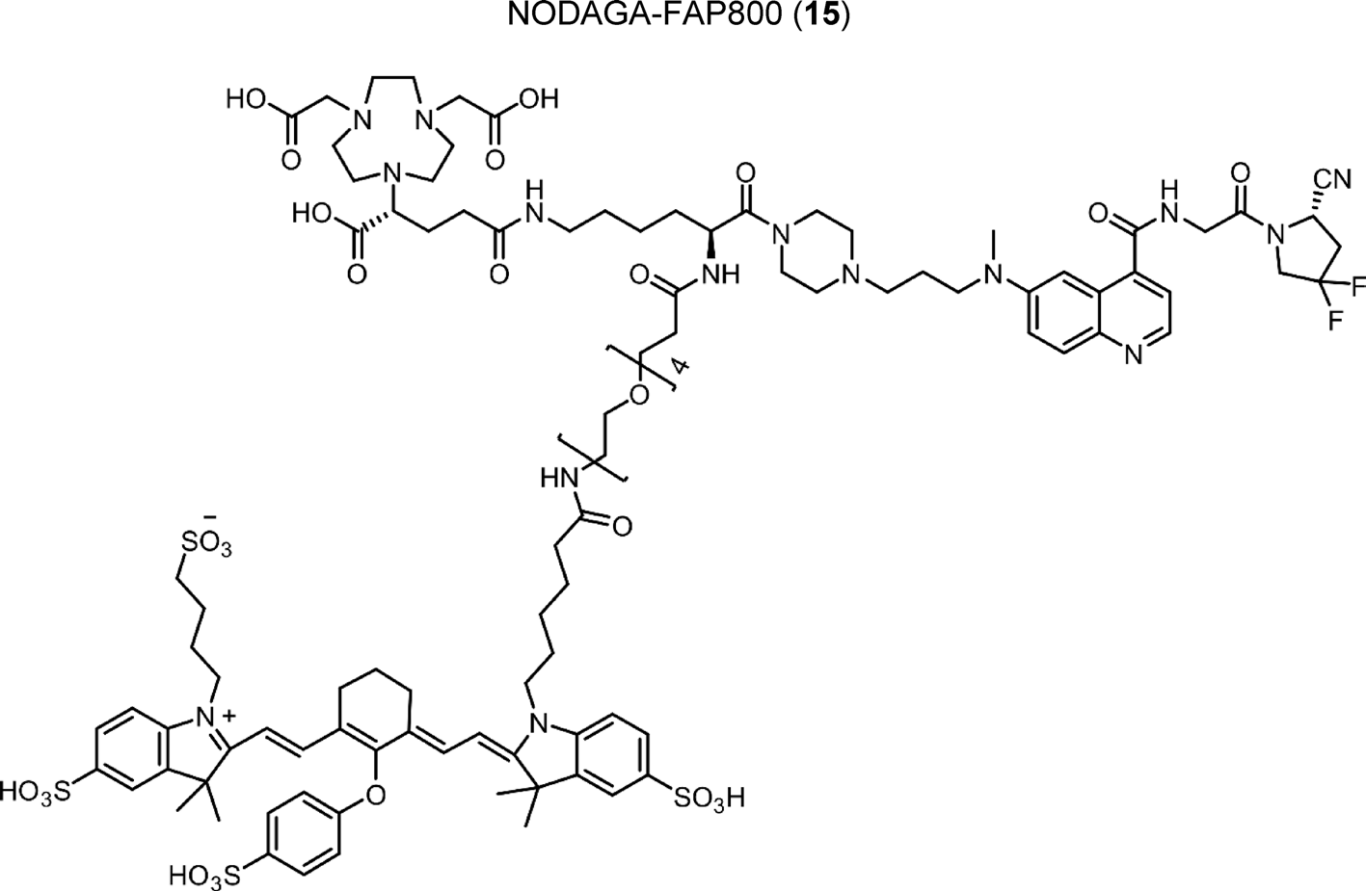


To a solution of IRDye800CW-NHS ester (1.0 mg, 0.857 µmol) and DIPEA (5.0 µL, 28.70 µmol) in 800 µL of DMF a solution of compound **13** (1.31 mg, 1.063 µmol) in 200 µL DMF was added and the reaction stirred at room temperature for 2 h. The solvent was removed under reduced pressure. The crude product was purified by preparative HPLC. The product was obtained as dark blue fluffy solid after lyophilization (0.99 mg, 0.449 µmol, 52%). Analytical HPLC: *t*_R_ = 17.79 min, HRMS (ESI^−^-ToF): calculated for C_103_H_139_F_2_N_15_O_29_S_4_: m/z = 1107.4301 [M-2H]^2−^, found m/z = 1107.4293 [M-2H]^2−^, The original HRMS spectrum is provided in the supplementary information. (Supplementary Fig. 6).

## Radiochemistry

### ^67^Ga radiosynthesis

^67^Ga was produced by the ^68^Zn(p,2n)^67^Ga reaction and purified as previously described (Kreller et al. 2024).

To a solution of [^67^Ga]GaCl_3_ in 0.05 M aqueous HCl, 0.5 M ammonium acetate buffer (pH 4.5) and ascorbic acid (1 g/mL) as a radioprotectant were added. The precursor (1 mM solution in DMSO) was added subsequently, and the mixture was incubated at 60 °C, while shaking at 750 rpm, for 30 min. The radiochemical conversion was determined by radio-TLC.

Quality control was performed by radio-HPLC analysis. The radiotracer was then either diluted with cell medium for in vitro testing or formulated by dilution with sterile 0.9% sodium chloride solution for animal experiments.

### ^68^Ga radiosynthesis

[^68^Ga]GaCl_3_ was eluted manually from a ^68^Ge/^68^Ga Generator* (*GalliaPharm®, Eckert & Ziegler, Berlin, Germany*)* with 5 mL of 0.1 M aqueous HCl solution. Thereby a fraction of 1.5 mL containing more than 90% of the radioactivity was collected and used directly for radiolabeling. A defined volume of the generator eluate was added to the reaction vial containing ascorbic acid (1 g/mL), which was serving as a radioprotectant. The pH of the solution was adjusted by the addition of 2 M ammonium acetate buffer to reach a final pH of 4.3. Afterwards, the precursor (1 mM solution in DMSO) was added and the mixture incubated at 95 °C for 5 min while shaking at 750 rpm. The RCC was then determined by radio-TLC or radio-UPLC.

Quality control was performed by radio-HPLC analysis. The radiotracer was then either diluted with cell medium for in vitro testing or formulated by dilution with sterile 0.9% sodium chloride solution for animal experiments.

### Radio-TLC

Radio-TLC analysis was conducted on Agilent iTLC-SA strips with 0.1 M citric acid buffer (pH 4) as mobile phase. TLC strips were analyzed using a BAS-1800 II instrument from Fujifilm (Tokio, Japan). Data was analyzed using Aida Image Analyzer software (Elysia-Raytest, Munich, Germany) version 4.27. A representative radio-TLC is provided in the supplementary information. (Supplementary Fig. 7).

### Radio-UPLC/ HPLC

Radio-HPLC analysis was performed on a modular LC-20 system (Shimadzu, Kyoto, Japan) consisting of two degassing units DGU-20A-3R and -5R, an LC30AD pump, a SIL-30AC autosampler, a CTO-20AC column oven, a CBM-20A communication bus module and an SPD-M30A diode array detector and a Gabi (Elysia, Angleur, Belgium) radioactivity detector with water + 0.1% trifluoroacetic acid (A) and acetonitrile + 0.1% trifluoroacetic acid (B) as mobile phase. For UPLC analysis a Phenomenex Kinetex C-18 (50 × 2.1 mm, 1.7 μM, 100 Å, gradient (A/B): *t*_0 min_ = 95/05 – *t*_0.3 min_ = 95/05 – *t*_4.5 min_ = 05/95 – *t*_5.5 min_ = 05/95 – *t*_6.0 min_ = 95/05 – *t*_7.5 min_ = 95/05, flow: 0.5 mL/min) and for HPLC analysis a Phenomenex Kinetex C-18 (250 × 4.6 mm, 5.0 μM, 100 Å, gradient: gradient (A/B): *t*_0 min_ = 95/05 – *t*_3.0 min_ = 95/05 – *t*_28.0 min_ = 05/95 – *t*_34.0 min_ = 05/95 – *t*_35.0 min_ = 95/05 – *t*_40.0 min_ = 95/05, 1.0 mL/min) column was used.

### In vitro affinity and kinetics

#### log ***D***_7.4_ determination

The n-octanol/ water partition coefficient was determined using the shake-flask method. Therefore, 10 µL of the ^68^Ga-labeled radioligand in aqueous buffer solution was added to a mixture of 990 µL of PBS (pH 7.4) and 1000 µL of n-octanol. The mixture was shaken at 800 rpm for 2 min, subsequently vortexed for 30 s and afterwards centrifuged. An aliquot of each phase was carefully withdrawn and the activity A was measured using a γ-counter. The measurements for each radioligand were performed in triplicate. The log *D*_7.4_ value was then calculated by the following equation.

$$ \log D = {\text{log }}\left( {\frac{{{\text{A}_{\text{n-octanol}}}}}{{{\text{A}_\text{PBS}}}}} \right) $$

#### Stability in human serum

Stability testing in human serum (Sigma Aldrich, Darmstadt, Germany) was carried out by incubation of the ^67^Ga-labeled radiotracer at 37 °C. After defined time points an aliquot was withdrawn and the serum proteins precipitated, by dilution with “supersol” (20% v/v ethanol, 5% v/v 5 mM EDTA, 0.5% v/v Triton X-100, 0.1% m/v saponin in aqueous solution). After centrifugation, the supernatant was analyzed by radio-HPLC.

#### Assessment of inhibitory constants (K_i_) for FAP and DPPIV via fluorometric enzyme inhibition assay

Affinity towards FAP was tested using a commercially available fluorometric assay. At the day of the experiment, all tested compounds were diluted in assay buffer (DPP assay buffer, BPS Bioscience, San Diego, CA, USA,) in a 1:1 dilution series, resulting in eleven concentrations, ranging from 2000 nM to 1.950 nM, equal to 200 nM to 0.195 nM per well. The substrate Ala-Pro-AMC (BPS Bioscience, concentration per well: 5 μM, *K*_m_ = 244 μM (Edosada et al. 2006)) was pipetted along with the inhibitor into a 96-well plate. Subsequently, the enzyme (hFAPα, recombinant protein) was dissolved in assay buffer and added to the wells. The final concentration of DMSO per well was 0.2%. The 96-well plate was immediately loaded into a multimode plate reader (Biotek Cytation 5, Agilent, Santa Clara, CA, USA). The fluorescence intensity for each well was measured over a time course of 30 min with measurement intervals of 15 s, an excitation wavelength of 360 ± 20 nm and an emission wavelength of 450 ± 20 nm. Data (relative fluorescence units, RFU) over the total measurement time was analyzed by linear regression. The slope of the resulting graphs was analyzed over the inhibitor concentration. The enzyme inhibition constant *K*_i_ was calculated using the Morrison equation for tight binding inhibitors (Williams and Morrison 1979). The active enzyme concentration was determined by extrapolating the linear front part (typically using inhibitor concentrations from 0.195 nM to 3.125 nM) of the dose–response curves to the x-axis intersection. The point of intersection represents the concentration of catalytically active enzyme, as described previously (Copeland 2013). All experiments were performed in triplicate.

The affinity towards DPPIV was determined using a commercially available inhibitor screening assay kit (Cayman Chemical, USA). The assay was performed according to the manufacturer’s instructions, and the data was analyzed as described above.

### Cell lines, culture and tissue preparation

HT1080 cells were chosen as cellular and xenograft models, due to their similarity to FAP expressing fibroblasts and its origin (fibrosarcoma). For that purpose, HT1080 wild-type cells (WT-HT1080; European Collection of Authenticated Cell Cultures, ECACC, United Kingdom) were used as control. Cells were cultured in DMEM with GlutaMax (#31966021, Gibco), supplemented with 10% v/v fetal bovine serum (#S0615 Sigma Aldrich, Germany), 1% penicillin/streptomycin (#15140–122, Gibco, USA), 1% Non-essential amino acids under normoxic (5% CO_2_; 37 °C) conditions. Upon reaching ~ 90% confluency, cells were passaged. Typically, cultures were discarded after ~ 20 passages. Overexpression of FAP was achieved through lentiviral transduction (hFAP-HT1080), as described and characterized previously (Loureiro et al. 2023). Following transduction, cells were treated identically to wild-type cells. Prior to in vitro experiments, cells were washed, trypsinized (0.05% Trypsin–EDTA, Gibco, USA), taken up in medium and an aliquot was counted using a CASY1 cell counter (Model TT, Schaerfe System, Reutlingen, Germany). For endpoint saturation binding, cells were diluted to 2.4–3.2 × 10^5^ cells/mL in medium and seeded to 48 well plates (Falcon Multiwell #353078, ThermoFisher, Germany) for typically 1–2 days. For real-time radioligand binding, typically 4.0 × 10^5^ cells/mL were seeded into one side of a petri dish (Nunclon, # 150350, ThermoFisher Germany) ~ 24–48 h before experiments. Tissue sections from hFAP-HT1080 xenografts (25 µm thick) were cut on a cryostat (Leica CM 1950; Leica, Germany) adhered to poly-l-lysine pretreated petri dishes. Depending on the tumor size, between 5 and 10 sections were placed in a ~ 20 mm circle, directly under the location of the detector. For the generation of xenografts, cells were harvested through trypsination, diluted to final number of cells (1.0–2.0 × 10^6^ WT/hFAP-HT1080 cells per animal), centrifuged and washed in PBS. Finally, the cells were taken up in fresh PBS (100 µL/animal) and stored briefly on ice.

#### Fluorescence cell imaging

WT/hFAP-HT1080 cells were seeded in an 18-chamber slide with 10,000 cells/well and cultured overnight. The medium was discarded and replaced by medium containing 1 µM of NODAGA-FAP647. The cells were then further incubated at 37 °C and 5% CO_2_. The binding of the ligand was stopped at the indicated time points by discarding the media and washing the cells twice with ice-cold PBS. The cells were then quickly fixed with 4% PFA/2.5% saccharose in PBS at 20 °C for 20 min. Following the fixation, cells were incubated with a 1:200 dilution of WGA-CF488A (Biotium #29022–1) in PBS to stain the cell membranes. Finally, the cell nuclei were stained with Hoechst33258 (Sigma #94403) diluted 5 µg/ml in PBS for 30 min at 20 °C. Cells were visualized with the Fluoview 1000 confocal laser scanning microscope (Olympus) using a 100 × oil immersion objective. Three representative images were captured for each well.

#### Saturation binding

Saturation binding (n ≥ 3, in triplicates) was performed to estimate thermodynamic dissociation constants (*K*_D_) and number of binding sites (*B*_max_). hFAP-HT1080 were cultured under the conditions described above. On the day of the experiment, 48-well plates were kept at 37 °C in an incubation chamber. The medium was replaced with 200 µL CO_2_-independent medium, (Gibco #18045088, ThermoFisher Germany) for total binding (TB) conditions. For the assessment of nonspecific binding (NSB), the same medium was used, containing 14 µM of FAPI-04 (in DMSO, resulting in 0.007% v/v). After 15 min preincubation for TB/NSB, 200 µL of each eight serial 1:1 dilutions (75 to 0.12 nM) of the respective radioligand were added (in triplicate), resulting in a final concentration per well of 35–0.06 nM. Cells were incubated with the respective radioligand for 60 min at 37 °C. The incubation medium was discarded and the wells washed with ice-cold PBS (2x ~ 30 s). The cells were then lysed with 500 µL 0.1 M NaOH + 1% SDS. For radioactivity detection, 400 µL of lysate was measured in a gamma counter (Perkin Elmer Wizard 1480). Additionally, binding to polystyrene in absence of cells was determined in 400 µL of lysate, along with the activity of all dilutions (50 µL). However, intrinsic plastic adherence of both compounds was very low. All counts were decay-corrected to a reference time (end of radiolabeling). Protein content was determined in an additional plate, subjected to the same conditions (pre-incubation, incubation and washing) by BCA assay and a mean value (µg/mL) used for this particular dataset (all replicate plates). From CPM measurements, final values (pmol/mg protein) were calculated using mean protein content and molar activity. Similarly, lysates from a random plate were subjected to fluorescent detection. For that purpose, 100 µL were pipetted bubble free into a transparent bottom 96 well imaging plate (Greiner Bio-One, µclear #655087). The plate was then imaged on a Typhoon 9500 bio imager (GE, USA) using either the Cy5 or IR-long filter-set (350–500 PMT voltage). From these images, fluorescence density (counts/mm^2^) was determined (Bio-Rad, Quantity One 4.6.8). All processed data was then subjected to non-linear iterative curve fitting (GraphPad Prism 10) to yield *B*_max_ (in pmol/mg protein or CNT/mm^2^) and *K*_D_ (in nM).

#### Real-time radioligand and fluorescence binding using LigandTracer™

To determine binding kinetics (association rate constant *k*_a_, dissociation rate constant *k*_d_, and thermodynamic dissociation constant *K*_D_), real-time assay systems (LigandTracer Yellow/White for radioactivity and Green with Red/NIR + and NIR +  + /IR detectors for fluorescence; Ridgeview Instruments AB, Sweden) were utilized. In a 100 mm petri dish, cells or tissue sections in medium (3–4 mL total volume), are located on one side, while the radioactive or fluorescent signal is detected on the opposite, elevated side. Rotation on an inclined base in set intervals (30 s) allows continuous measurement of signal in the target (cells/tissue) against background area (no cells/tissue). Experiments were performed at room temperature, using CO_2_-independent medium, usually with 0.5–1% w/v bovine serum albumin, (#1ETA, Carl Roth, Germany). Association was determined through incubation with two distinct concentrations (between 1–5 and 4.5–10 nM, 60 min each, additive) for each compound. For that purpose, the same ligand preparation was typically employed for radioactive and fluorescent detection. This was followed by replacement with fresh medium and observation of dissociation for at least 2 h. Additionally, the dissociation rate constant was further investigated beyond 6 h, either using fluorescent detection or via gallium-67 (t_1/2_: 78.3 h). This approach provides reliable kinetic measurements in a 1:1 interaction setting, as previously shown (Onell and Andersson 2005).

At the end of each experiment, dishes were inspected for cell detachment, which was not observed for short (2–3 h) dissociation experiments. Longer (usually overnight) experiments showed beginning live cell detachment at ~ 6 h.

For the acid-wash experiments, the two association phases were carried out as described above. Afterwards, the medium containing the tracer was replaced by 50 mM glycine buffer (pH 2.8) for 8 min with data acquisition continuing. Thereafter, the glycine buffer was removed, cells carefully washed with medium, and fresh medium added. The dissociation phase followed.

Binding data was evaluated using TraceDrawer (1.9.2, Ridgeview Instruments AB, Sweden). Traces were imported, potential spikes (> 100% sudden signal increases over previous datapoints) removed and each trace was normalized to its own baseline (= 0%) and highest value (= 100%, typically at the end of the second association phase). This allows comparing data across experiments and ultimately global fitting, without differences, e.g. of molar activity, affecting it. Typically, at least 3 individual traces per ligand, substrate and modality were recorded. Data was fitted in TraceDrawer using a 1:1 interaction model, with/without accounting for bulk effect. A 1:1 interaction is the simplest model, as it describes the binding of a ligand at a constant concentration to the target, with each one molecule of ligand binding to one molecule of target.

### Xenografting and in vivo PET and fluorescence imaging

Female athymic NMRI-nude mice (Rj:NMRI-Foxn1nu, Janvier Labs, France or Charles River, Germany) between 8 and 20 weeks of age were used. Subsequently, the prepared cells (1.0–2.0 × 10^6^ hFAP/WT-HT1080 per animal) were subcutaneously injected into the right and left thigh, respectively. Xenograft tissue for real-time binding experiments was derived from animals injected with both target-positive and negative cells, however only showing growth of one tumor entity. When approaching endpoints, mice were anesthetized and euthanized. The tumor was quickly removed, frozen in − 45 °C 2-methylbutane and stored in sealed tubes at − 80 °C.

PET and X-ray computed tomography (CT) were performed in a dedicated small animal scanner (nanoScan PET/CT, Mediso, Hungary). Animals received a single dynamic scan corresponding to 0–2 h post injection. The radiotracer solution (8 – 20 MBq injected activity) was delivered via an i.v. catheter (lateral tail vein) and PET acquisition was started simultaneously with the injection. Blocking was performed by pre-injection (~ 5 min prior to tracer) of 100 nmol of FAPI-04. Three-dimensional list mode data were binned (400–600 keV energy window) and sorted into 35-time frames (15 × 10 s, 5 × 30 s, 5 × 60 s, 4 × 300 s, 3 × 600 s, 3 × 1200 s). Time frames were then reconstructed using the Tera-Tomo™ 3D algorithm with a voxel size of 0.4 mm and corrected for decay, scatter, and attenuation. PET data was analyzed using Rover (ABX GmbH, Germany). Three-dimensional volumes of interest (VOI) were delineated based on organs/tissues [xenografts, kidney, liver, shoulder and muscle (~ biceps brachii)] and a fixed threshold (35% of the measured maximum intensity) was applied. This yielded standardized uptake values (SUV = [MBq detected activity/mL tissue] / [MBq injected activity/g body weight], mL/g) for the chosen VOI.

Optical imaging was performed using a multimodal IVIS-CT (Perkin Elmer, USA). Compounds were injected [0.09 mg/kg (n = 1)/0.33 mg/kg (*n* = 3) for NODAGA-FAP647; 0.17 mg/kg (*n* = 3) for NODAGA-FAP800, diluted in 150 µL of sterile 0.9% NaCl solution] into the lateral tail vein, either directly or via catheter. Fluorescence imaging started ~ 2 min post injection for 2–3 h, with 1 min, 3–5 min and 10 min intervals, along with a white-light image. Additional scans were performed after 24 h and, if applicable, later. The fluorescence of the respective fluorophore was captured (NODAGA-FAP647: *λ*_Ex/Em_ = 640/680 nm, NODAGA-FAP800: *λ*_Ex/Em_ = 745/800 nm). Emitted fluorescence intensity was analyzed (Perkin Elmer, Living Image 4.7.4) in raw data images.

### Statistics

Time-dependent in vivo tracer uptake/binding in target-positive/negative subcutaneous tumors (either from PET imaging as SUVmean or optical imaging as fluorescence intensity) was subjected to statistical testing (Two-Way ANOVA) using GraphPad Prism 10. Factors were tracer uptake/binding across the two tumor entities (hFAP-positive/negative) and time. This provides information on whether tracer uptake/binding differs significantly between the target-positive and negative tumors and over time, independent of each other (main effect). A significant interaction indicates that tumor uptake/binding depends on time after injection.

## Supplementary Information

Below is the link to the electronic supplementary material.

Supplementary Material 1

## Data Availability

Additional data is available in the supplement and are available from the corresponding author upon reasonable request.

## Abbreviations

5-ALA: 5-Aminolevulinic acid
*B*_max_: Maximum number of specific binding sites
CAFs: Cancer-associated fibroblasts
CT: Computed tomography
DIPEA: *N,N*-Diisopropylethylamine
DPPIV: Dipeptidyl peptidase 4
ESI: Electrospray ionization
FAP: Fibroblast activation protein
FAPI: Fibroblast activation protein inhibitor
hpi: Hours post injection
HPLC: High-performance liquid chromatography
HRMS: High resolution mass spectrometry
IC_50_: Half-maximal inhibitory concentration
ICG: Indocyanine green
*k*_a_: Association rate constant
*K*_D_: Thermodynamic dissociation constant
*k*_d_: Dissociation rate constant
*K*_i_: Thermodynamic inhibition constant
LC-MS: Liquid chromatography-mass spectrometry
MB: Methylene blue
mpi: Minutes post injection
MRI: Magnetic resonance imaging
NIR: Near-infrared
NMR: Nuclear magnetic resonance
NODAGA: 1,4,7-Triazacyclononan-1-glutaric acid-4,7-diacetic acid
PBS: Phosphate-buffered saline
PEG: Polyethylene glycol
PET: Positron emission tomography
PFA: Paraformaldehyde
PSMA: Prostate-specific membrane antigen
RCC: Radiochemical conversion
RFU: Relative fluorescence unit
ROI: Region of interest
SPECT: Single-photon emission computed tomography
TLC: Thin-layer chromatography
TME: Tumor microenvironment
ToF: Time-of-flight
UPLC: Ultra-performance liquid chromatography
VOI: Volume of interest
WGA: Wheat germ agglutinin
