# Supplementary material for: Synthesis and preclinical evaluation of FAP-targeting radiotracers for PET and optical imaging
Source: EJNMMI Radiopharm Chem. 2025 Dec 5;10:77. doi: 10.1186/s41181-025-00398-9 (PMC12686243; doi:10.1186/s41181-025-00398-9)
Supplement: Supplementary file 1 — Supplementary Material 1 [file 41181_2025_398_MOESM1_ESM.docx]

# Synthesis and Preclinical Evaluation of FAP-Targeting Radiotracers for PET and Optical Imaging

Jürgen Kogler^1,2^, Cornelius K. Donat^1^, Johanna Trommer^1,2^, Klaus Kopka^1,2,3,4^, Sven Stadlbauer^1,2^*

ORCID: Jürgen Kogler: 0009-0005-4996-7399, Cornelius Donat: 0000-0003-3750-3579, Sven Stadlbauer: 0000-0003-2276-5330, Klaus Kopka: 0000-0003-4846-1271

^1^Institute of Radiopharmaceutical Cancer Research, Helmholtz-Zentrum Dresden-Rossendorf (HZDR), D-01328 Dresden, Germany.

^2^Technische Universität Dresden, School of Science, Faculty of Chemistry and Food Chemistry, D-01062 Dresden, Germany.

^3^German Cancer Consortium (DKTK), Partner Site Dresden, and German Cancer Research Center (DKFZ), Heidelberg, D-69120, Germany.

^4^National Center for Tumor Diseases (NCT), NCT/UCC Dresden, a partnership between DKFZ, Faculty of Medicine and University Hospital Carl Gustav Carus, TUD Dresden University of Technology, and Helmholtz-Zentrum Dresden-Rossendorf (HZDR), 01307 Dresden, Germany.

*corresponding author: Sven Stadlbauer, s.stadlbauer@hzdr.de

# Synthesis schemes

**Supplementary Fig. 1** Synthesis route of intermediate **13**

**Supplementary Fig. 2** Synthesis of radiolabeling precursors NODAGA-FAP647 **14** and NODAGA-FAP800 **15**

# Analytical HPLC and HRMS chromatograms


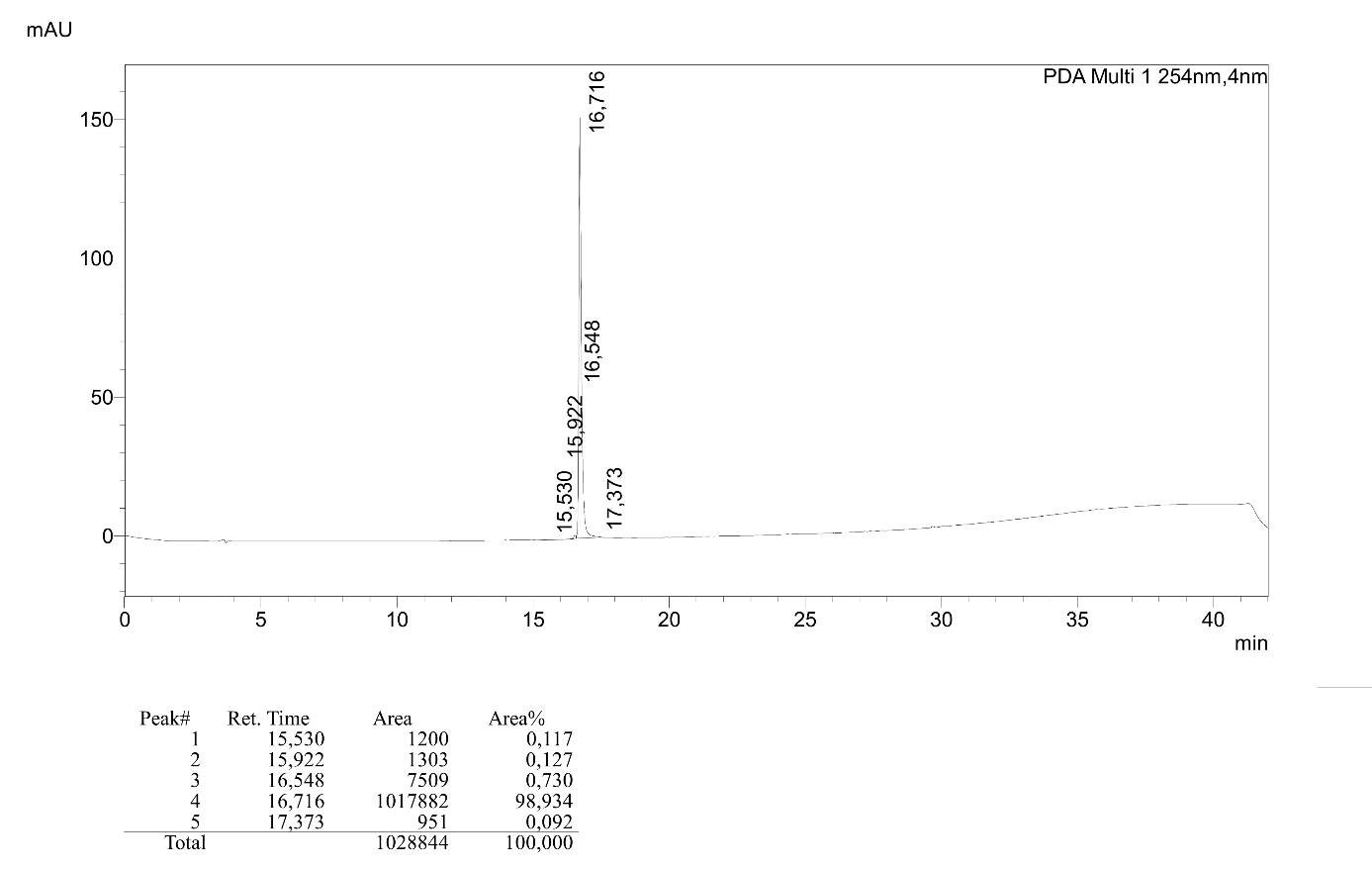


**Supplementary Fig. 3** HPLC Chromatogram of NODAGA-FAP647 (**14**), detection of absorbance at 254 nm


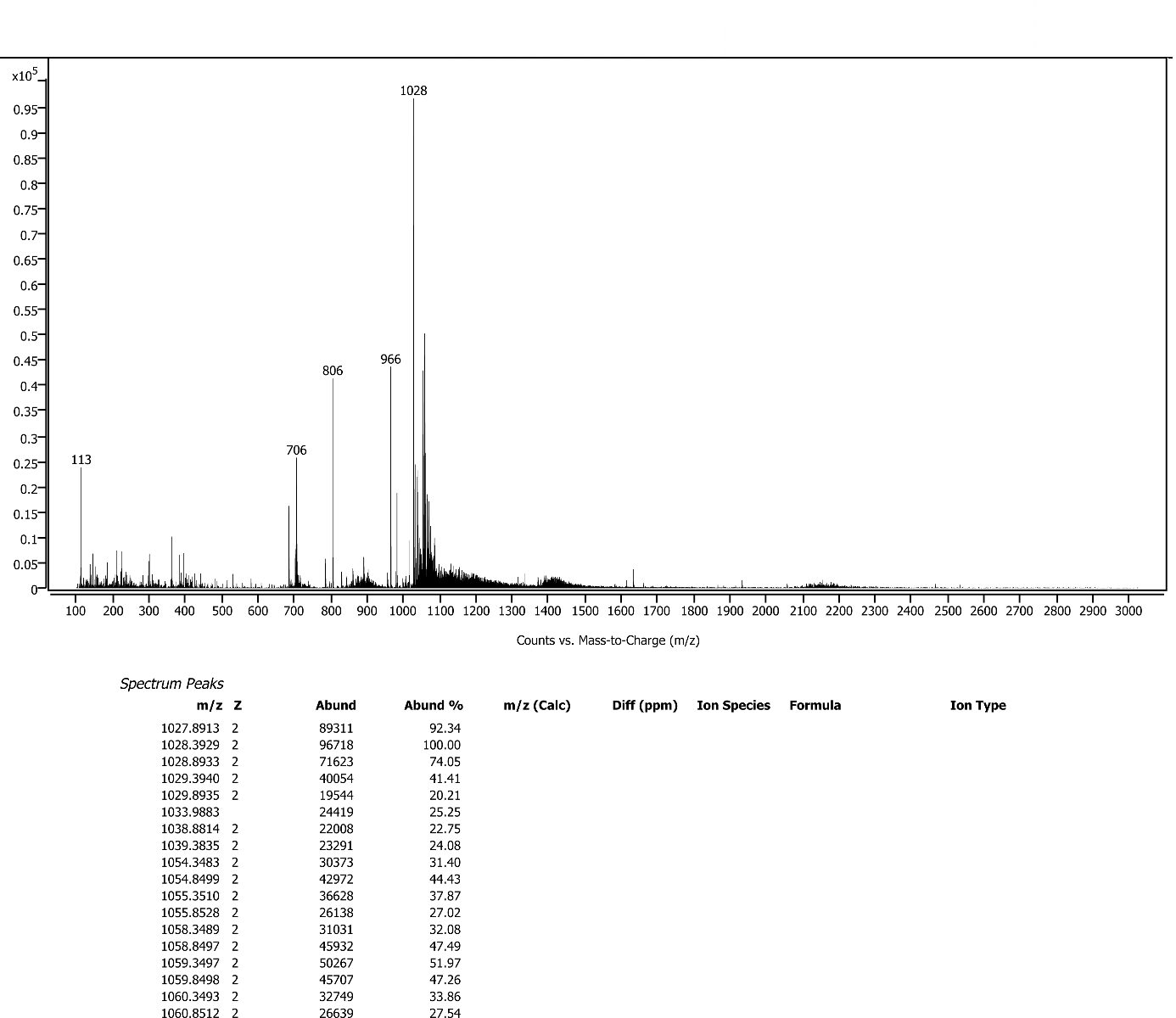


**Supplementary Fig. 4** ESI^-^-ToF high resolution mass spectrometry chromatogram of NODAGA-FAP674 (**14**)


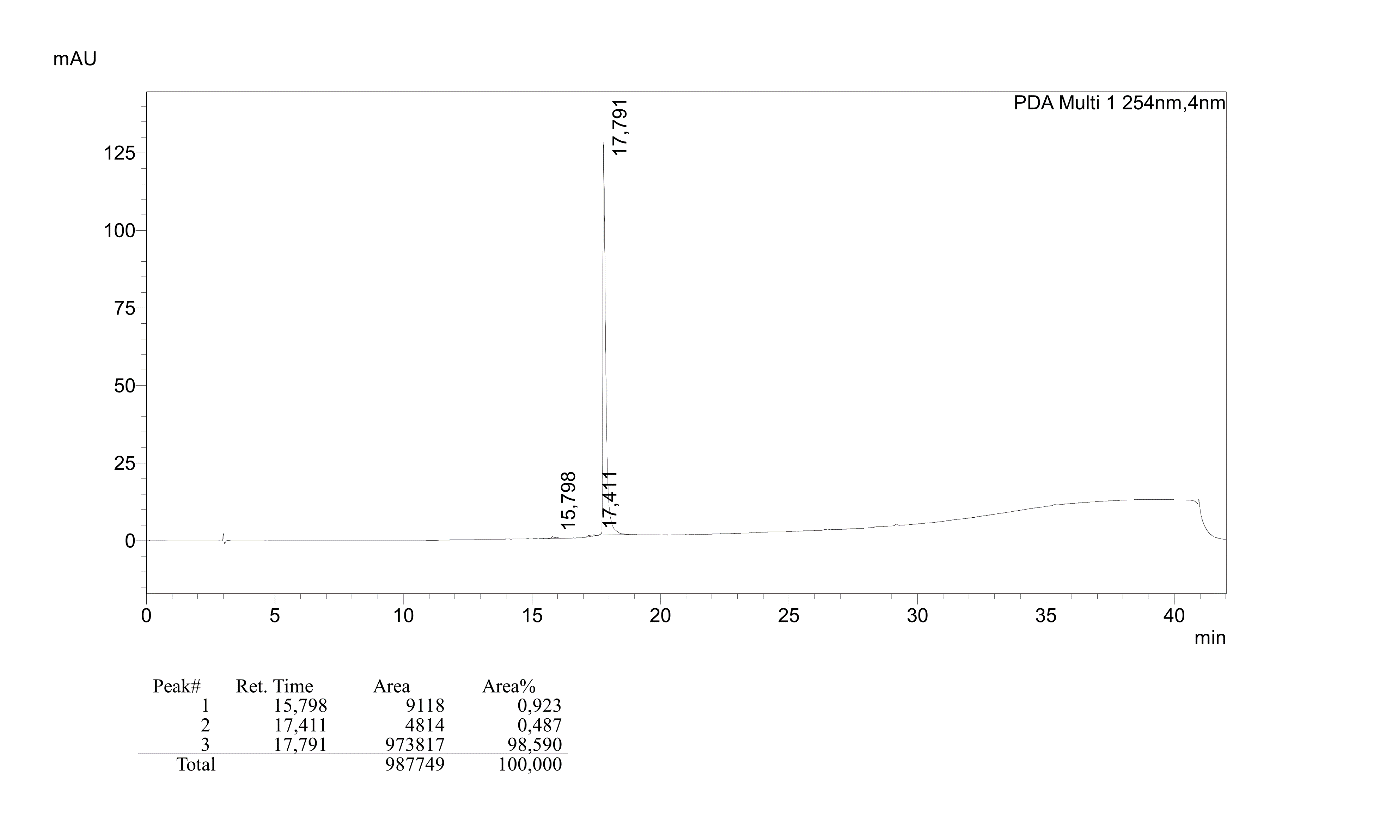


**Supplementary Fig. 5** HPLC Chromatogram of NODAGA-FAP800 (**15**), detection of absorbance at 254 nm


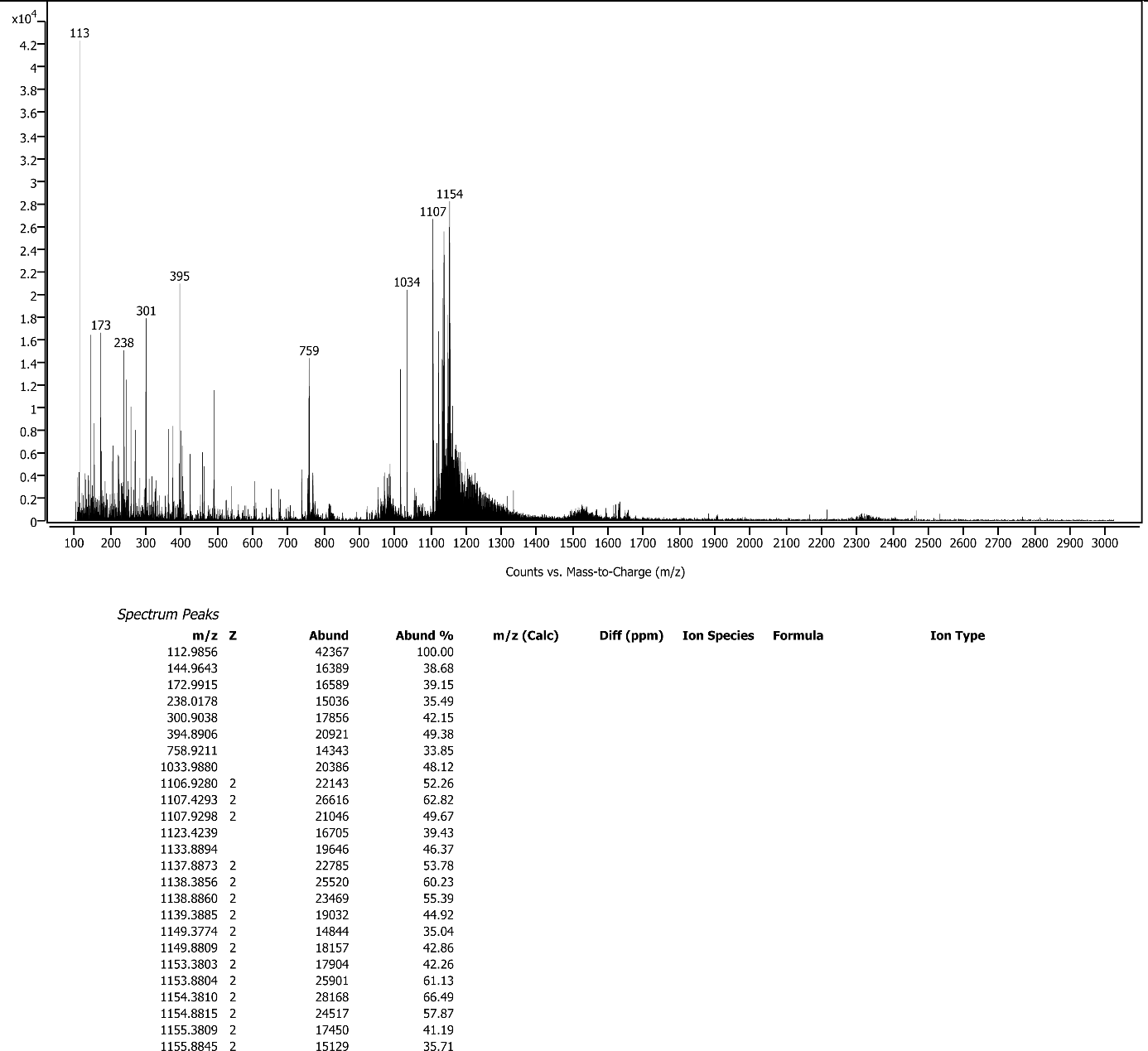


**Supplementary Fig. 6** ESI^-^-ToF high resolution mass spectrometry chromatogram of NODAGA-FAP800 (**15**)


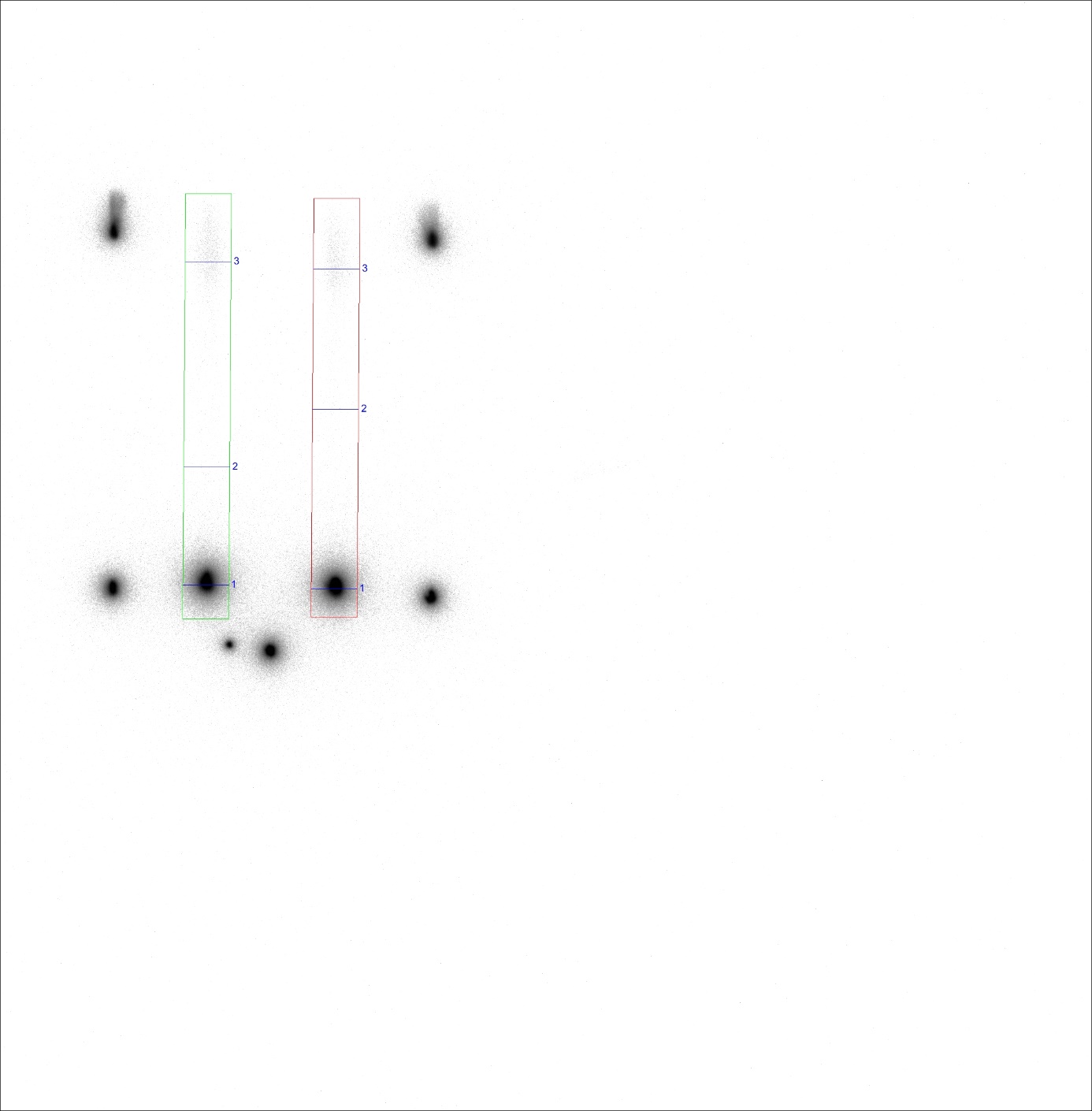


| Compound | Spot | Integral | Integral - background | Integral - background (%) |
| --- | --- | --- | --- | --- |
| [^67^Ga]Ga-NODAGA-FAP647 | 1 | 30215 | 29570 | 98.2 |
| [^67^Ga]Ga-NODAGA-FAP647 | 3 | 1180 | 535 | 1.80 |
| [^67^Ga]Ga-NODAGA-FAP800 | 1 | 27078 | 26535 | 99.2 |
| [^67^Ga]Ga-NODAGA-FAP800 | 3 | 746 | 203 | 0.77 |

**Supplementary Fig. 7** Representative radio-TLC of a reaction control of ^67^Ga-labeling of [^67^Ga]GaNODAGA-FAP647 (left lane) and [^67^Ga]GaNODAGA-FAP800 (right lane), spot 1 represents the radiolabeled radiotracer at the baseline of the TLC strip (R_f_ = 0.0-0.1), spot 3 represents uncomplexed [^67^Ga]Ga^3+^ (R_f_ = 0.9-1.0)


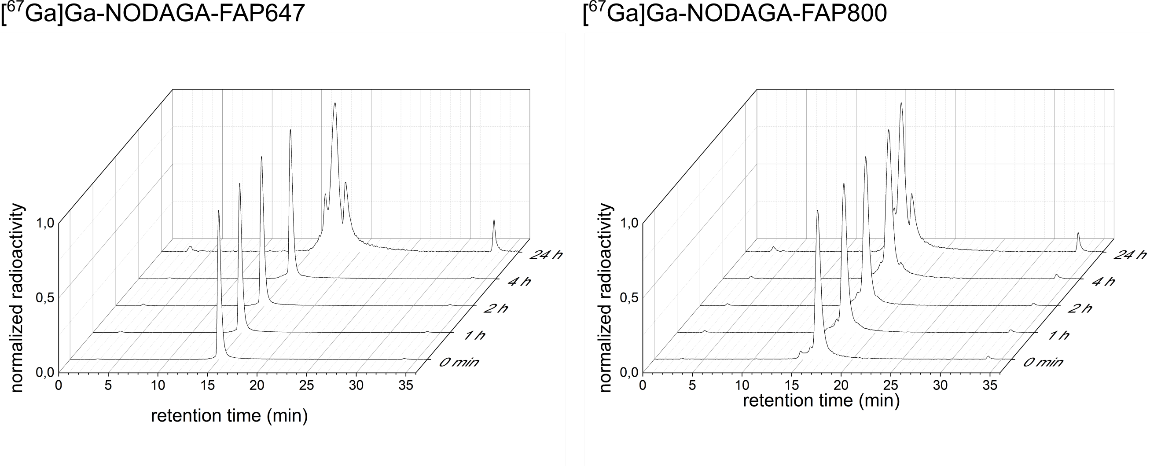


**Supplementary Fig. 8** Radio-HPLC chromatograms of [^67^Ga]Ga-NODAGA-647 (left) and [^67^Ga]Ga-NODAGA-FAP800 (right) from stability testing in human serum at 37 °C, no degradation was observed up to four hours of incubation

**Supplementary Fig. 9** Inhibition of recombinant hFAP with varying concentrations of NODAGA-FAP647, turnover of fluorogenic substrate Ala-Pro-AMC measured at the emission wavelength 450 ± 20 nm and analyzed by linear regression

**Supplementary Fig. 10:** Inhibition of human recombinant dipeptidylpeptidase IV with sitagliptin as positive control (**a**), NODAGA-FAP647 (**b**) and NODAGA-FAP800 (**c**) as a single experiment, which was performed in duplicate. Black line represents fit by non-linear regression analysis equation for IC_50_, Values are mean ± SD.

# Real-time binding


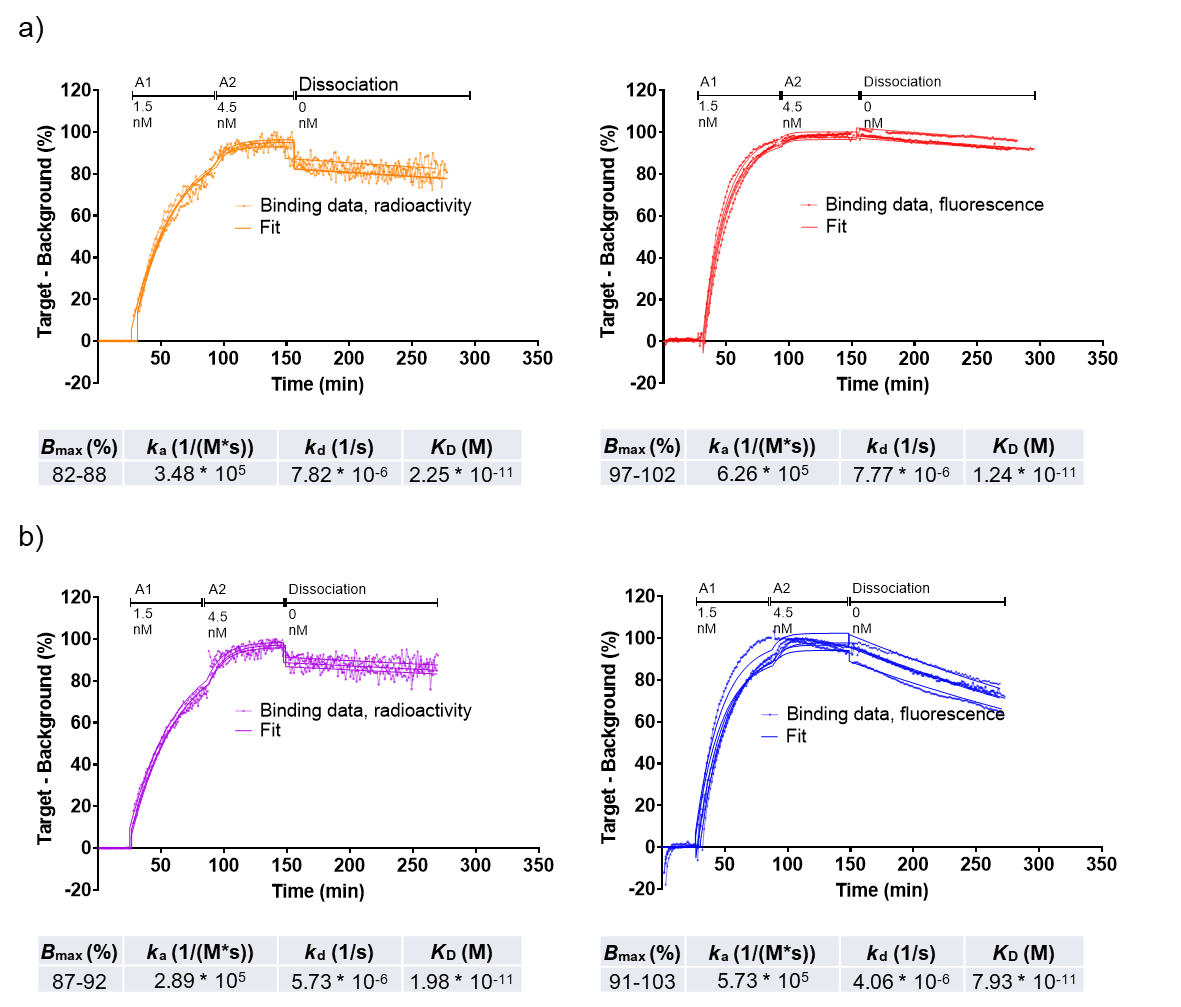


**Supplementary Fig. 11** Real-time binding of [^68^Ga]Ga-NODAGA-FAP647 (**a**) and [^68^Ga]Ga-NODAGA-FAP800 (**b**) to hFAP-HT1080 cells, detected via radioactive (left-side graph) or fluorescent (right-side graph) label, along with fitted kinetic parameters.

Each trace was normalized to baseline (0%) and highest value (typically at the end of the second association phase, 100%). This approach allows global fits without signal intensity differences.

Colored lines with symbols (red/orange: fluorescent/radioactive label detection of [^68^Ga]Ga-NODAGA-FAP647; blue/purple: fluorescent/radioactive label detection of [^68^Ga]Ga-NODAGA-FAP800) represent individual traces (actual binding, signal of target − background area in %; each decay or fluorescence corrected), with fitted data indicated by respective colored solid lines. Kinetic constants were fitted over all traces (global), while *B*_max_ was fitted per binding curve (local). Data from n = 3-4 independent experiments.


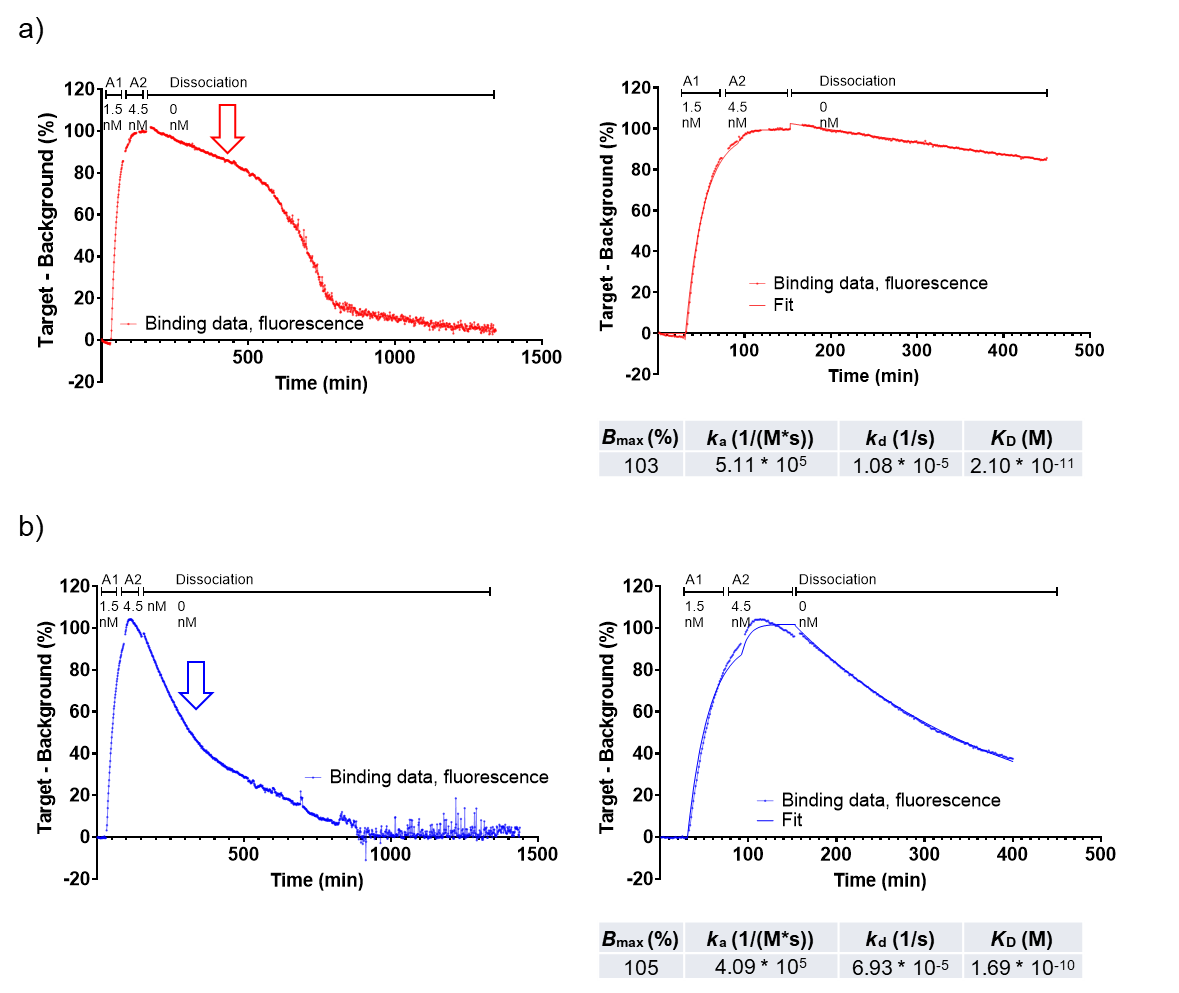


**Supplementary Fig. 12** Real-time binding of NODAGA-FAP647 (**a**) and NODAGA-FAP800 (**b**) to hFAP-HT1080 cells with a dissociation phase >10 hours

Left side graphs show dissociation over the total time. The red (**a**) and blue (**b**) arrows indicate beginning of substantial cell detachment. Right side graph shows data with dissociation period restricted to 450 min, along with fitted kinetic parameters.

Colored lines with symbols (red/blue: fluorescent label detection of NODAGA-FAP647/ NODAGA-FAP800; represent individual traces (actual binding, signal of target − background area in %; fluorescence corrected), with fitted data indicated by respective colored solid lines.

Each trace was normalized to baseline (0%) and highest value (typically at the end of the second association phase, 100%). Data from n = 1 experiment, using either radioactive or fluorescent label detection.


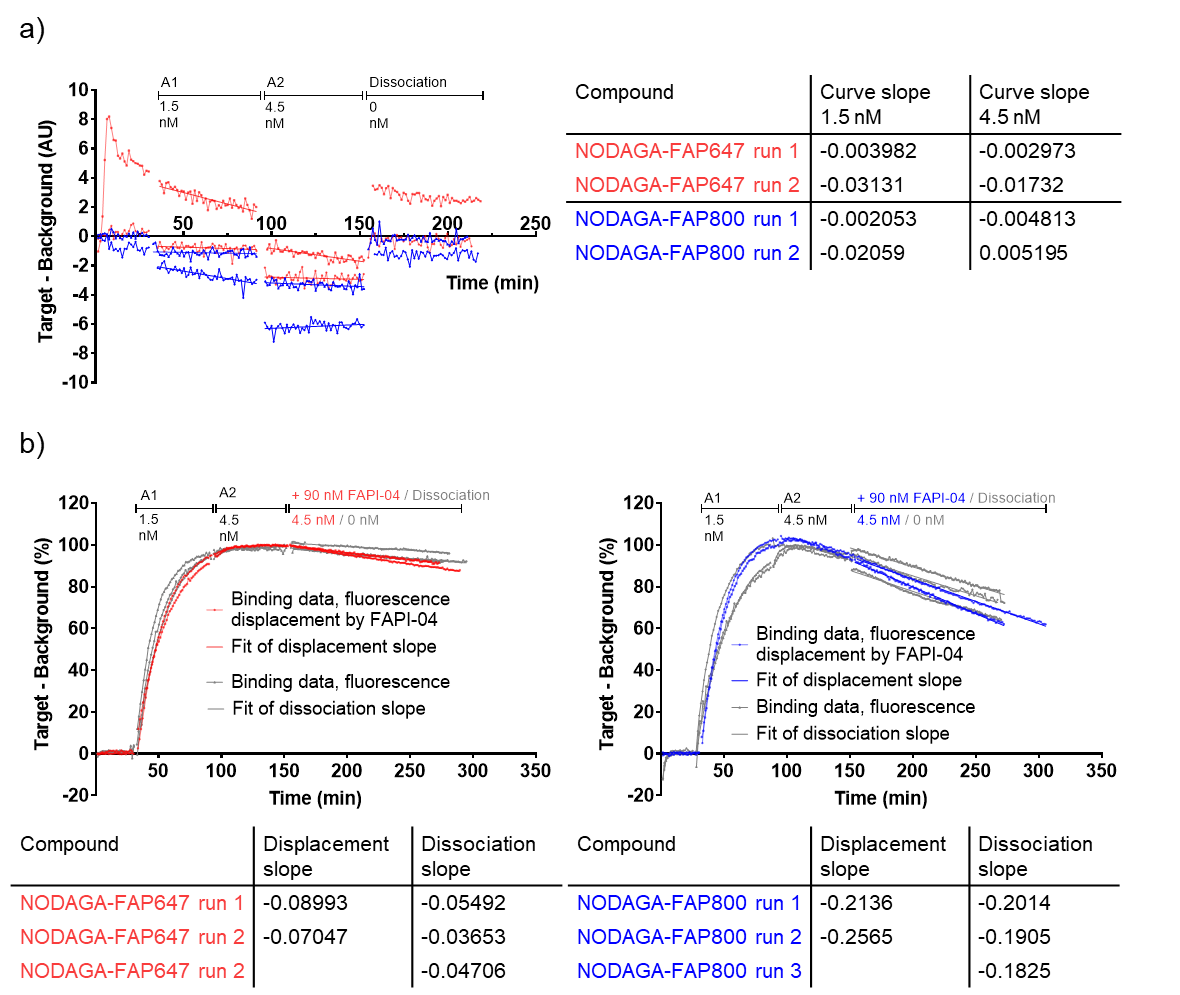


**Supplementary Fig. 13** Control experiments confirming target specificity of NODAGA-FAP647/800

(**a**) Fluorescent label detection of NODAGA-FAP647 (red) or NODAGA-FAP800 (blue) binding to WT-HT1080 cells. Data confirms target specificity of both compounds, as indicated by lack of signal increase (slopes close to zero).

(**b**) Fluorescent label detection of NODAGA-FAP647 (red, left-side graph) or NODAGA-FAP800 (blue, right-side graph) binding to hFAP-HT1080 cells, followed by displacement via unlabeled FAPI-04. Following both association phases, an excess (20x, 90 nM) of FAPI-04 was added, resulting in a displacement of the labeled tracer following the same pattern as observed for dissociation (grey lines).

Colored lines with symbols (red/blue: fluorescent label detection of NODAGA-FAP647/ NODAGA-FAP800) represent individual traces [actual binding, signal of target − background area in AU (**a**) or % (**b**); fluorescence corrected], with fitted data indicated by respective colored solid lines. Data from n = 2-3 independent experiments.


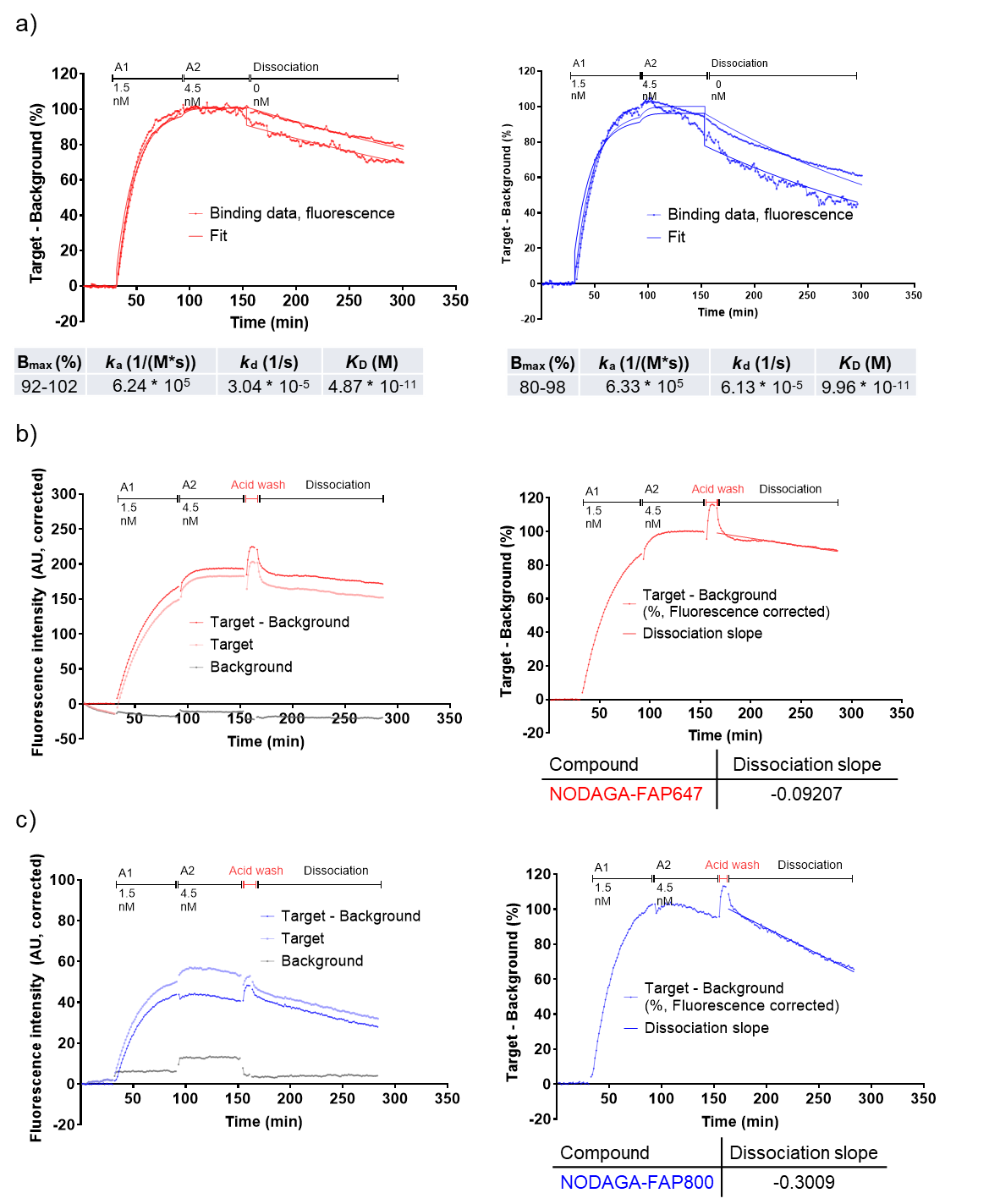


**Supplementary Fig. 14** Control experiments confirm that highly stable binding rather than internalization is responsible for slow dissociation of NODAGA-FAP647/800.

(**a**) Fluorescence label detection of NODAGA-FAP647 (left-side graph) and NODAGA-FAP800 (right-side graph) binding to PFA-fixed hFAP-HT1080 cells. Data confirms slow dissociation instead of internalization.

Colored lines with symbols (red/blue: fluorescent label detection of NODAGA-FAP647/NODAGA-FAP800) represent individual traces [actual binding, signal of target − background area in %. Data from n = 2 independent experiments.

(**b**) Fluorescence label detection of NODAGA-FAP647 binding to hFAP-HT1080 cells, followed by an acid wash (glycine buffer pH 2.8, 8 min).

(**c**) Fluorescence label detection of NODAGA-FAP800 binding to hFAP-HT1080 cells, followed by an acid wash (glycine buffer pH 2.8, 8 min).

Signal increase during the acid wash is likely due to the change in pH affecting quantum yield. Afterwards, the glycine buffer was exchanged for medium and dissociation phase followed.

Colored lines with symbols (red/blue: fluorescent label detection of NODAGA-FAP647/ NODAGA-FAP800) represent individual traces [actual binding, signal of target − background area in AU or %. Data from n = 1 experiment.

# PET imaging

**Supplementary Fig.** **15** Time-activity curves (0-2 hpi) of uptake of [^68^Ga]Ga-NODAGA-FAP647 (left) and [^68^Ga]Ga-NODAGA-FAP800 (right) into hFAP-HT1080 and WT-HT10180 tumors. Two-way ANOVA reveals a significantly main effect of tumor entity (hFAP/WT-HT1080 tumors), time and interaction of tumor entity and time.

| **VOI** | **AUC (95% CI)**  **[^68^Ga]Ga-NODAGA-FAP647** | **AUC (95% CI)**  **[^68^Ga]Ga-NODAGA-FAP800** |
| --- | --- | --- |
| hFAP-HT1080 | 170.6 (144.2 – 197.0) | 130.3 (123.0 – 137.6) |
| WT-HT1080 | 48.84 (43.31 – 54.38) | 50.47 (45.41 – 55.53) |
| Kidney | 107.3 (103.8 – 11.9) | 118.6 (114.3 – 122.8) |
| Liver | 40.77 (39.54 – 42.01 | 53.47 (51.98 – 54-97) |
| Joint | 66.49 (58.42 – 74.56) | 71.85 (64.22 – 79-47) |
| Muscle | 30.54 (27.12 – 33.97) | 31.56 (27.21 – 35.90) |

**Supplementary Table 1** Area under the curve values derived from time-activity curves of [^68^Ga]Ga-NODAGA-FAP647 and [^68^Ga]Ga-NODAGA-FAP800 .


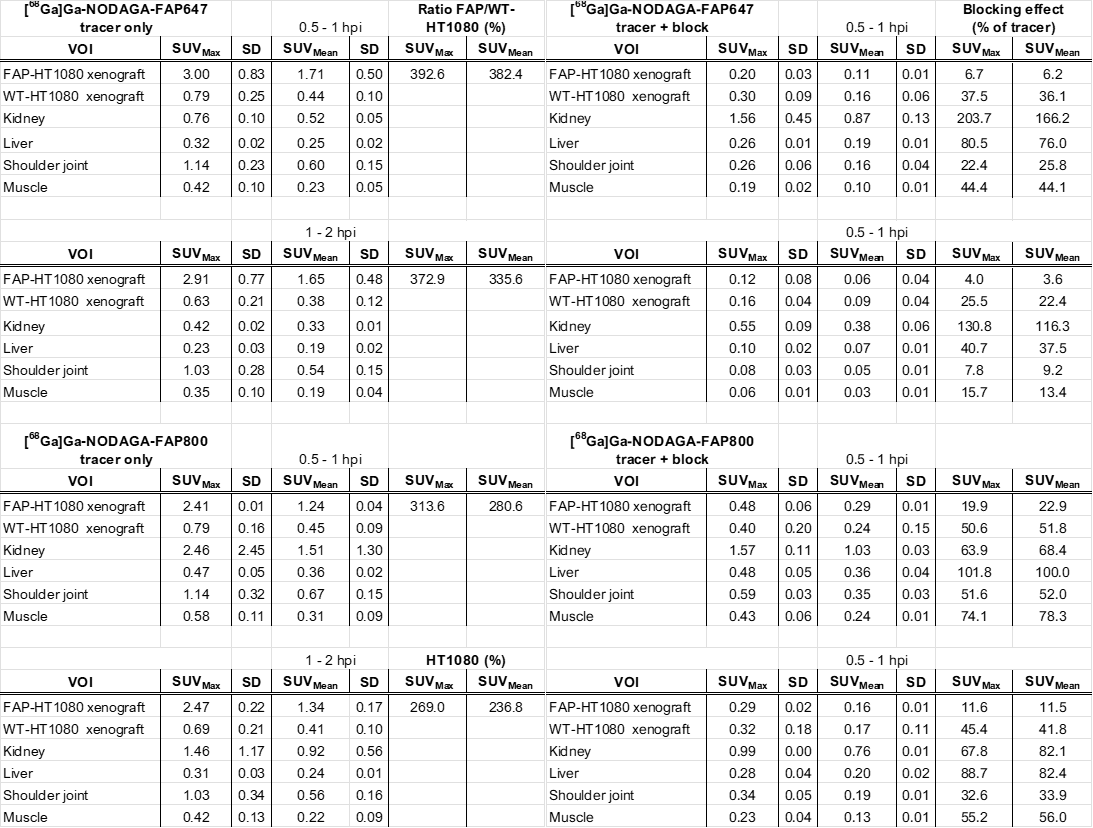


**Supplementary Fig. 16** Small animal PET imaging of [^68^Ga]Ga-NODAGA-FAP647 and [^68^Ga]Ga-NODAGA-FAP800 in selected volumes of interest (VOI) of female NMRI-nude mice. The data shows standardized uptake values (SUV_MAX_ and _MEAN_) of tracer (left column block) and tracer+block (right column block; 100 nmol FAPI-04, injected 5 min prior to tracer). For animals injected with tracer only, contrast of target-positive (hFAP-HT1080) vs. target-negative (WT-HT1080) tumor are provided via ratios. Similarly, SUV of animals injected with tracer+block vs. tracer only provides the blocking effect. SUV data is mean from n=3 for animals injected with tracer only and n=2 for animals injected with tracer +block. Standard deviation (SD) is given in adjacent columns.

***In vivo* fluorescence imaging shows sufficient contrast between target-positive and target-negative tumors and normal tissue**

Fluorescence intensity (average radiant efficiency), as displayed in Figure 5/6B, was used to plot various ratios over time for NODAGA-FAP647 (Supplementary Fig. 16a) and NODAGA-FAP800 (Supplementary Fig. 16b). Ratios were calculated for:

1. hFAP- vs. WT-HT1080 tumor (dark red line with circles) for NODAGA-FAP647/800
2. hFAP-HT1080 tumor vs. muscle (light red line with triangles)
3. hFAP-HT1080 tumor vs. tailbase (light red line with rhombi)
4. WT-HT1080 tumor vs. muscle (dark blue line with squares)
5. WT-HT1080 tumor vs. tailbase (light blue line with circles).

These plots demonstrate a sufficient signal contrast between target-positive and target-negative tumors for both NODAGA-FAP647 and NODAGA-FAP800. While an overall similar trajectory was observed for both compounds, NODAGA-FAP647 yielded a higher hFAP- vs. WT-HT1080 and hFAP-HT1080 vs. muscle ratio across the first 120 minutes. Both compounds exhibited a very stable WT-HT1080 vs. muscle ratio close to one.


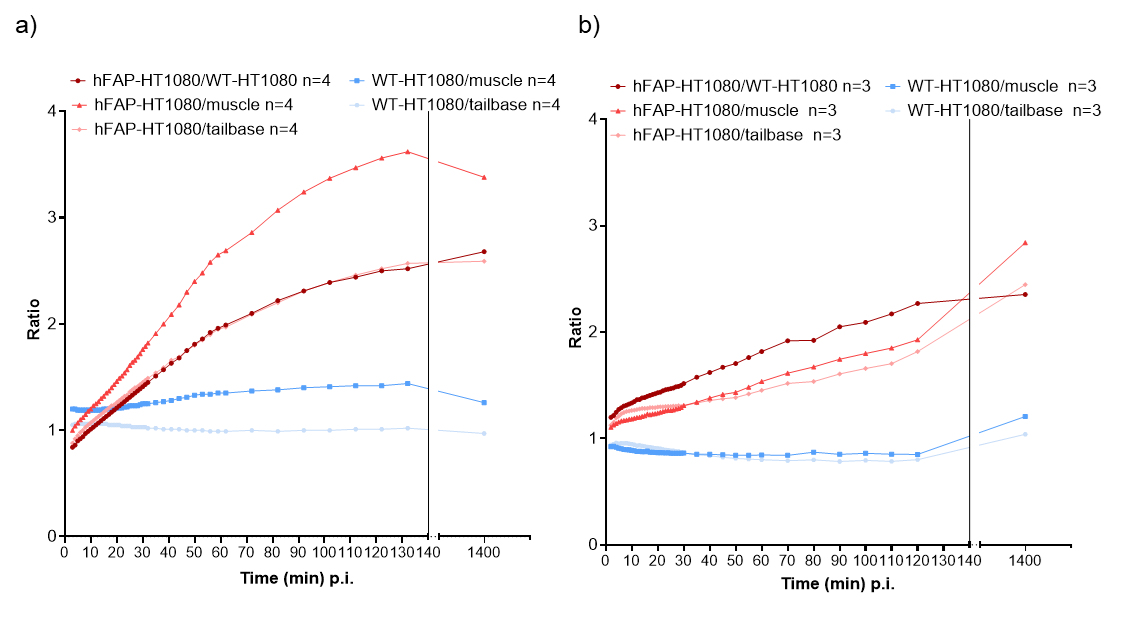


**Supplementary Fig.** **17** In vivo specificity of the fluorescence signal for NODAGA-FAP647 (**a**) and NODAGA-FAP800 (**b**). Fluorescence intensity (average radiant efficiency) expressed as ratio of different ROIs (tumors, muscle, tailbase). Ratios of: hFAP-H1080 vs. WT-HT1080 (dark red line with circles), hFAP-HT1080 vs. muscle (red line with triangles), WT-HT1080 vs. muscle (dark blue line with squares), hFAP-HT1080 vs. tailbase (light red line with rhombi) and hFAP-HT1080 vs. tailbase (light blue line with circles).

***In vivo* fluorescence imaging of NODAGA-FAP800 shows hFAP-HT1080 tumor retention beyond 24 hours post injection**

Initial experiments with NODAGA-FAP800 indicated elevated signal in the hFAP-HT1080 tumor beyond 24 hours post injection (hpi), which was then qualitatively confirmed in an additional animal. While overall fluorescence beyond 24 hpi was much lower compared to the first 2 hours, the target-positive tumor still showed a higher fluorescent signal compared to the target-negative tumor.


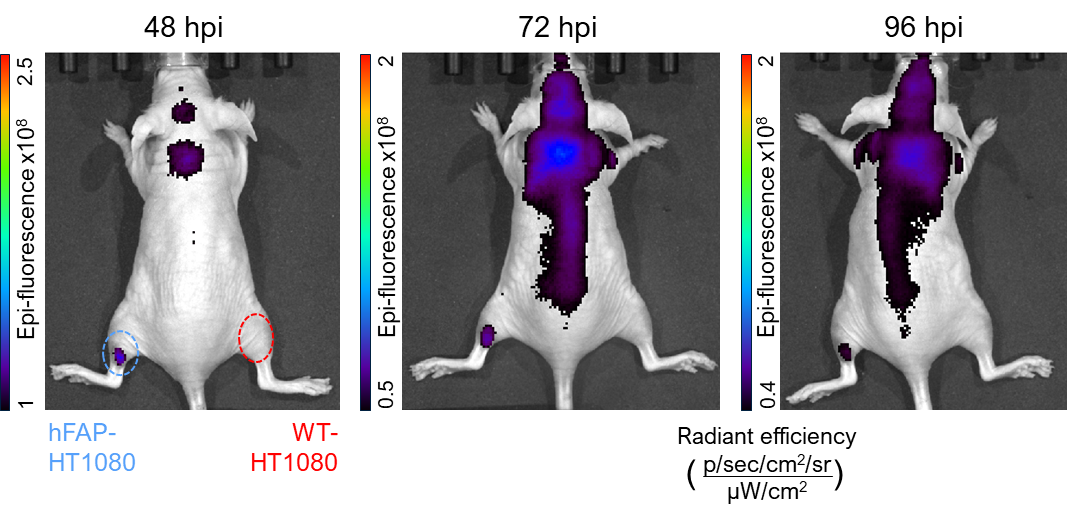


**Supplementary Fig. 18** Example fluorescence images (from radiant efficiency, [p/s/cm²/sr] / [µW/cm²]) of NODAGA-FAP800 in nude mice carrying hFAP/WT-HT1080 xenografts (left/right thigh) at later timepoints. Animal was injected with 0.19 mg/kg (n=1) and imaged 48-72 hours post injection (hpi). Scaling differs across all images.

# *Ex vivo* radioactive/fluorescence imaging in frozen sections shows co-localization of radioactive and fluorescent signal.

To demonstrate co-localization of the radioactive and fluorescence signal, autoradiography and fluorescence imaging was performed on tissue sections *ex vivo*. Following a PET scan with [^68^Ga]Ga-NODAGA-FAP647, the animal was euthanized under anesthesia by cervical dislocation. Injected activity for this particular animal was 12.76 MBq (decay corrected), corresponding to 80.44 µg/kg (1.11 nmol). The hFAP- and WT-HT1080 tumors were excised and frozen in ~-45°C cold 2-methyl butane. From mid-tumor onwards, 12 µm thick sections were cut on a Leica CM 1950 cryostat and mounted on glass slides (Super-Frost+; Menzel, Germany). Muscle tissue (biceps brachii) served as control. Imaging was performed on a Typhoon 9500 bio-imager (GE, USA) at 25 µm pixel size. Fluorescence was imaged at ~155 min post injection (pi) using the Cy5 laser/filterset. At ~195 min pi, slides were exposed to BAS-SR imaging plates (GE) for 4 hours and subsequently scanned with the phosphor imaging laser/filterset. Both images were scaled according to visibility, with scaling matched for hFAP/WT-HT1080 tumors and exported as .tif files. Using FIJI, grey values were assigned a color (photostimulated luminescence=red; fluorescence=blue) and registered using a rigid approach, followed by merging. In the hFAP-HT1080 tumors, an identical distribution pattern of the radioactivity and fluorescence signal was observed (Supplementary Fig. 17, upper row). Using the same scaling, only very little signal from radioactivity/fluorescence could be observed in the target-negative tumor (Supplementary Fig. 17, lower row). This further supports the target specificity of [^68^Ga]Ga-NODAGA-FAP647.


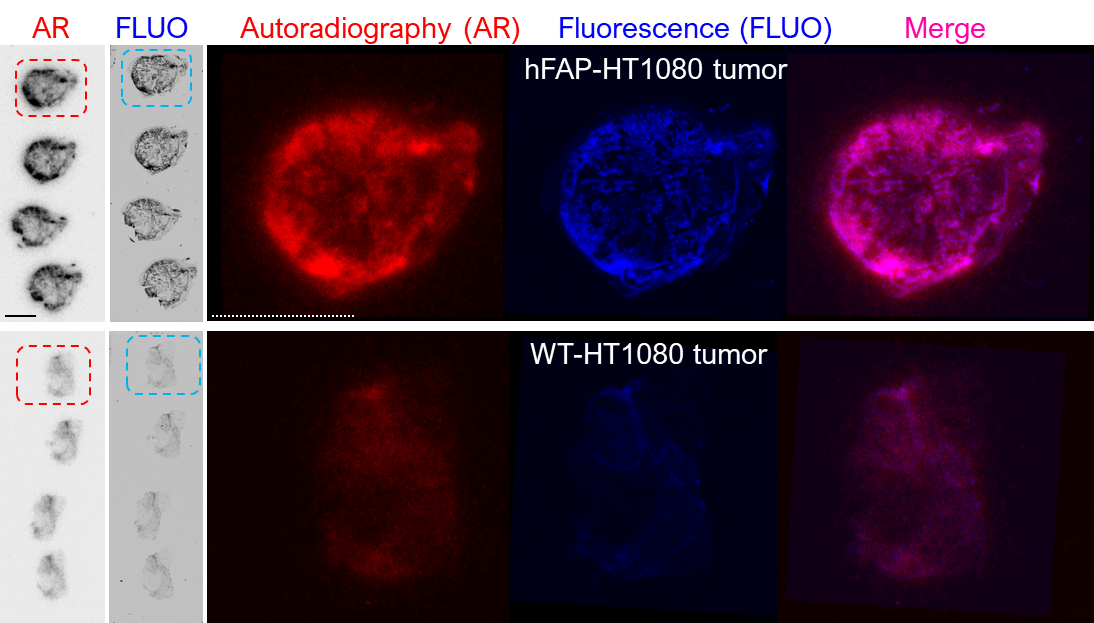


**Supplementary Fig. 19** Autoradiography/fluorescence imaging of 12 µm mid-tumor sections derived from excised tumors directly after PET imaging, showing overlapping radioactive and fluorescent signal. The hFAP-HT1080 tumor sections are displayed in the upper panel, while WT-HT1080 tissue is shown in the lower panel. Fluorescence imaging was performed starting ~155 and autoradiography ~195 min post injection. Scaling is matched for both modalities. Solid black/dotted white line corresponds to 5 mm.

# References

1. Jansen K, Heirbaut L, Verkerk R, Cheng JD, Joossens J, Cos P, et al. Extended structure-activity relationship and pharmacokinetic investigation of (4-quinolinoyl)glycyl-2-cyanopyrrolidine inhibitors of fibroblast activation protein (FAP). J Med Chem. 2014;57:3053-74. doi:10.1021/jm500031w.

2. Loktev A, Lindner T, Burger EM, Altmann A, Giesel F, Kratochwil C, et al. Development of Fibroblast Activation Protein-Targeted Radiotracers with Improved Tumor Retention. J Nucl Med. 2019;60:1421-9. doi:10.2967/jnumed.118.224469.

3. Lindner T, Loktev A, Altmann A, Giesel F, Kratochwil C, Debus J, et al. Development of Quinoline-Based Theranostic Ligands for the Targeting of Fibroblast Activation Protein. J Nucl Med. 2018;59:1415-22. doi:10.2967/jnumed.118.210443.

4. Kreller M, Brühlmann SA, Knieß T, Kopka K, Walther M. Production of Medical Radionuclides in the Center for Radiopharmaceutical Tumor Research—A Status Report. Instruments. 2024;8. doi:10.3390/instruments8010010.

5. Loureiro LR, Hoffmann L, Neuber C, Rupp L, Arndt C, Kegler A, et al. Immunotheranostic target modules for imaging and navigation of UniCAR T-cells to strike FAP-expressing cells and the tumor microenvironment. J Exp Clin Cancer Res. 2023;42:341. doi:10.1186/s13046-023-02912-w.

6. Edosada CY, Quan C, Wiesmann C, Tran T, Sutherlin D, Reynolds M, et al. Selective inhibition of fibroblast activation protein protease based on dipeptide substrate specificity. J Biol Chem. 2006;281:7437-44. doi:10.1074/jbc.M511112200.

7. Williams JW, Morrison JF. The kinetics of reversible tight-binding inhibition. Methods in Enzymology: Academic Press; 1979. p. 437-67.

8. Copeland RA. Tight Binding Inhibition. Evaluation of Enzyme Inhibitors in Drug Discovery; 2013. p. 245-85.

9. Onell A, Andersson K. Kinetic determinations of molecular interactions using Biacore--minimum data requirements for efficient experimental design. J Mol Recognit. 2005;18:307-17. doi:10.1002/jmr.745.
